# Smaller Genetic Risk in Catabolic Process Explains Lower Energy Expenditure, More Athletic Capability and Higher Prevalence of Obesity in Africans

**DOI:** 10.1371/journal.pone.0026027

**Published:** 2011-10-10

**Authors:** Cheng Xue, Yun-Xin Fu, Yuhai Zhao, Yun Gong, Xiaoming Liu

**Affiliations:** 1 Human Genetics Center, The University of Texas Health Science Center at Houston, Houston, Texas, United States of America; 2 GuangDong Institute for Monitoring Laboratory Animals, Guangdong, China; 3 Laboratory for Conservation and Utilization of Bio-resources, Yunnan University, Yunnan, China; 4 The University of Texas Health Science Center at Houston, Medical School, Houston, Texas, United States of America; Fred Hutchinson Cancer Research Center, United States

## Abstract

Lower energy expenditure (EE) for physical activity was observed in Africans than in Europeans, which might contribute to the higher prevalence of obesity and more athletic capability in Africans. But it is still unclear why EE is lower among African populations. In this study we tried to explore the genetic mechanism underlying lower EE in Africans. We screened 231 common variants with possibly harmful impact on 182 genes in the catabolic process. The genetic risk, including the total number of mutations and the sum of harmful probabilities, was calculated and analyzed for the screened variants at a population level. Results of the genetic risk among human groups showed that most Africans (3 out of 4 groups) had a significantly smaller genetic risk in the catabolic process than Europeans and Asians, which might result in higher efficiency of generating energy among Africans. In sport competitions, athletes need massive amounts of energy expenditure in a short period of time, so higher efficiency of energy generation might help make African-descendent athletes more powerful. On the other hand, higher efficiency of generating energy might also result in consuming smaller volumes of body mass. As a result, Africans might be more vulnerable to obesity compared to the other races when under the same or similar conditions. Therefore, the smaller genetic risk in the catabolic process might be at the core of understanding lower EE, more athletic capability and higher prevalence of obesity in Africans.

## Introduction

Performance difference due to racial difference can be clearly observed in certain fields. For example, track and field sports are dominated by African athletes with the fact that most world records, such as the men's and women's 100-meter dash, 200-meter dash, 400-meter dash, etc. are held by them (http://en.wikipedia.org/wiki/List_of_world_records_in_athletics). Some possible factors, including genetic, physical, and metabolic factors, have been discussed and examined for a long time [1], but this is still an open question scientifically.

In public health, it was observed that the energy consumption (Energy expenditure, EE) of each standardized activity, such as lying, sitting and standing, was significantly higher in Europeans than in Africans [2]. And further observations showed that in routine activities, such as sleeping, resting, and free-living, EE was all lower in Africans than in Europeans [3]–[6]. Hunter et al (2000) reported that African-descendant women had lower aerobic fitness than European-descendant women, suggesting that lower EE in African-descendent women was mediated by lower volumes of metabolically active mass [7]. And Pan et al (2009) reported that obesity was more prevalent in African-American than in European-American [8]. Therefore it was hypothesized that differences in energy efficiency may contribute to the higher prevalence of obesity in African descendents than in Europeans [9]. But there is a fundamental question: why is EE smaller in Africans than in Europeans?

Many factors might be involved in more athletic capability and higher prevalence of obesity in Africans, such as biological, psychological and sociological factors in nature [1], [8]. In this study we tried to explore the genetic mechanism underlying lower EE in Africans, and proposed a framework to explain the racial difference in athletic capability and the prevalence of obesity.

During the transmission of genetic materials from generation to generation, some mutations occur, which in turn change amino acid, so called missense mutations or non-synonymous substitutions. In some cases these mutations may render the resulting protein to be nonfunctional, and are harmful to the function of genes. Recently, the harmful effects of missense mutations (deactivating the gene or affecting the gene function) have been estimated in PolyPhen-2 (Polymorphism Phenotyping v2) [10]. PolyPhen-2 is a tool that predicts possible impact of a missense mutation on the function of a human protein using straightforward physical and comparative considerations (annotations in UNIPROT/SWISS-PROT, change of electrostatic charge, change of hydrophobicity, and change of secondary structure, etc) [10]. In this study, a group of missense mutations with possibly harmful impact on genes involved in the catabolic process were greatly focused. For simplicity, the alleles of the targeted SNPs (single nucleotide polymorphism) with minor allele frequency (MAF) were called mutations through this article.

Energy is generated in the catabolic process. As we know, in most cases, the loss function on a gene will not cause apparent damage on an individual's health, majorly due to the complementary role of genetic robustness. But the accumulation of mutations might impair this genetic robustness [11]–[14]. Therefore, accumulating harmful mutations may increase the genetic risk in the catabolic process. Based on this hypothesis, we screened 231 common variants with possibly harmful impact on 182 genes out of total 1601 genes engaged in the catabolic process. And then we calculated the total number of mutations (Num) and the sum of the harmful probabilities on these variants (R) as two indices of the genetic risk in the catabolic process at population level, followed by the statistical analyses of association among human populations. Results showed that most of Africans had significantly smaller genetic risk in the catabolic process than Europeans and Asians, which might cause higher efficiency of generating energy in Africans. In sports, especially in athletic competitions, athletes need great amount of energy expenditure in a short period of time. Higher efficiency of energy generation might help make African-descendent athletes more powerful. In routine activities, higher efficiency in generating energy might cause consuming smaller amount of body mass. Therefore Africans might be more likely to be obese when under the same or similar conditions.

## Results

Genotype data in HapMap project was used in this study. Eleven selected human groups and their sample numbers are listed in Table 1. Because the size of each population group was usually small in Hapmap data, the SNPs with MAF>0.05 were mainly focused. Eleven human groups were historically organized and expressed as sets.
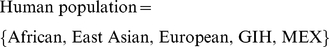
(1)In which African = {ASW, LWK, MKK, YRI}, East Asian = {CHB, CHD, JPT} and European = {CEU, TSI}. The subsets of African, East Asian, and European were termed as human subpopulations in our study. GIH and MEX were two independent groups.

**Table 1:** 11 human groups sampled in Hapmap Data Realease #3 and their sample numbers.

Subpopulation	Group name	Group symbol	Sample number (n)
European	Utah residents with Northern and Western European ancestry from the CEPH collection	CEU	165
	Toscans in Italy	TSI	102
Asian	Han Chinese in Beijing, China	CHB	137
	Chinese in Metropolitan Denver, Colorado	CHD	109
	Japanese in Tokyo, Japan	JPT	113
African	African ancestry in Southwest USA	ASW	87
	Luhya in Webuye, Kenya	LWK	110
	Maasai in Kinyawa, Kenya	MKK	184
	Yoruba in Ibadan, Nigeria	YRI	203
Independent groups	Gujarati Indians in Houston, Texas	GIH	101
	Mexican ancestry in Los Angeles, California	MEX	86

Out of 1601 known genes involved in the catabolic process, 182 genes with common harmful mutations (MAF>0.05 and r_i_>0.2) were screened, where r_i_ was the harmful probability of mutation for i^th^ SNP and was estimated by using Polyphen-2 (see Methods). Table S1 lists the detailed information of these 182 genes. For all the SNPs on 182 genes, the SNPs with the “benign” effect (r_i_<0.2) were excluded, leaving 231 SNPs entering the final analyses (details of the processing are shown in Table 2). Table S2 shows the detailed information of these 231 SNPs, including chromosome information, groups, gene symbol, and values of r_i_. Table S3 lists the minor allele frequency (MAF) of 231 screened SNPs for each of 11 studied population groups.

**Table 2:** The count numbers of non-synonymous SNPs and their genes screened in catabolic process in human groups.^a^

Human groups	Total		MAF>0.05 and r_i_>0.2		Group-specific^b^ (MAF>0.05 and r_i_>0.2)	
	No. SNPs	No. Genes	No. SNPs	No. Genes	No. SNPs	No. Genes
ASW	882	508	139	119	39	38
CEU	808	489	123	110	10	10
CHB	929	536	107	90	9	9
CHD	688	429	108	89	10	10
GIH	741	453	120	103	-	-
JPT	797	484	113	98	15	15
LWK	1030	571	128	108	28	26
MEX	983	563	124	108	-	-
MKK	814	473	136	114	36	35
TSI	824	496	132	115	19	19
YRI	853	497	128	112	28	28
Total (non-redundancy)	1361	676	231	182	115	102

a MAF is minor allele frequency, and r_i_ is the harmful probability of SNP estimated in Polyphen-2 (trained by HumDiv dataset). r_i_>0.2 indicates that the variant is possibly harmful. GIH and MEX are independent groups, so the numbers of their group-specific SNPs are not shown.

b Group-specific indicates that the SNPs considered are not shared in human groups and subpopulations.

### Hereditability of mutations in the catabolic process

Of the 231 SNPs screened, 48 were shared among all 11 population groups (population-shared SNPs), 52 were shared among African subpopulations, 50 were shared among East Asian subpopulations, and 65 were shared among European subpopulations (see Table S2). The patterns of MAF distribution on population-shared SNPs among different population groups indicated that the closer the historical relationships among groups were held, the more similar the MAF distribution patterns were observed (see Figure S1, S2 and S3). ASW, LWK, MKK and YRI were correlated genetically to each other. Therefore the patterns of MAF distribution were quite similar among them, but apparently differed from other subpopulations (European and East Asian) or independent groups (GIH and MEX). These observations suggested that first, mutations on these population-shared SNPs originated from heredity, and their heritability might be strong; second, after the historical splits of human groups these mutations were still being retained among all human groups regardless of environmental changes and/or genetic drift. Therefore they were more likely to be under natural selection.

### Association between the harmful probability of a SNP (r_i_) and its MAF

**Figure 1:**
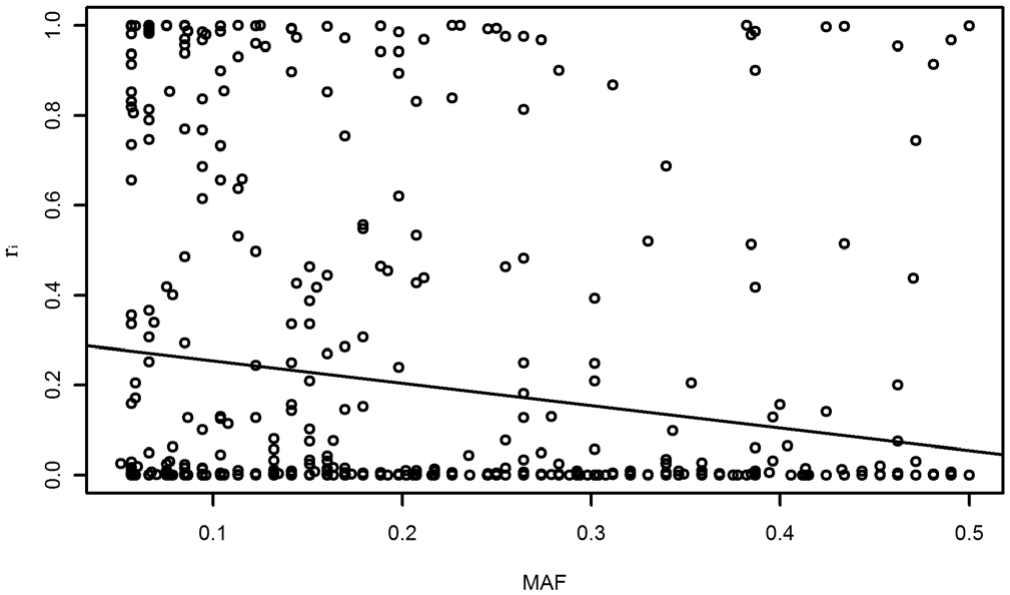
Correlation between MAF and the harm probability of SNPs (r_i_) on common SNPs in ASW. MAF is minor allele frequency. The equation of the line of best fit is f(x) = −0.4993x+0.3046, where x is MAF, correlation coefficient r^2^ = 0.03304, and P = 2.177
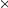
10^−5^.

In principle, due to negative selection, the frequency of a mutation is inversely proportional to the magnitude of its harmful effect [15]. In reality, this proportional relationship might be indistinct because other evolutionary forces, such as positive selection, migration, and genetic drift, are also involved. We assessed the association between r_i_ and MAF for SNPs considered in this study. In ASW, the MAF of a SNP was showed to be negatively and weakly associated with its harmful probability (r^2^ = 0.03304) and the association was significant with *P_Value* 2.177
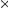
10^−5^ (see Figure 1). We also got the similar results for other studied groups such as CEU, CHB, and CHD, etc (data not shown). These observations supported the theoretical prediction that association existed between the harmful probability of a SNP (r_i_) and its MAF [15].

### Permutation results

As we know, different groups have different genetic backgrounds such that one group could have more genetic variants than another group naturally. In order to reduce the background noise level in assessing the actual genetic risk, permutation test was carried out. Results showed that both R and Num were significantly different among subpopulations and groups (see Table 3 and Figure S4). ASW and MKK (African) had significantly more mutations and larger genetic risk than CEU and TSI (European) (P<0.01). Especially, R and Num in ASW (African-American) were larger than those in CEU (European-American) by about 2.6% and 2.8%, respectively. TSI in European had significantly larger R and Num than East Asian (P<0.05), while CEU had larger R and Num, but with no significance (P>0.05). Additionally, we also performed another permutation test to re-sample randomly 231 SNPs (the number of SNPs screened from the catabolic process) from 3357 SNPs screened on 18161 human genes in human genomes. And similar results were obtained (see Figure S5). The results in these two permutation tests suggested that in the background R and Num in African were usually larger or not smaller than European and East Asian.

**Table 3:** Analysis of Variance for Observations (ANOVA) of genetic risk estimated by R and Num on genes re-sampled randomly in human genomes.^a^

Index		Df	Sum Sq.	Mean Sq.	F value	Pr(>F)
R	Subpopulation	2	278	138.898	15.723	1.57 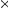 10^−7^ ***
	group	6	924	154.07	17.44	<2.2 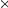 10^−16^ ***
	Residuals	4491	39675	8.834		
Num	Subpopulation	2	4354	2177.17	37.62	<2.2 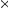 10^−16^ ***
	group	6	6402	1066.92	18.436	<2.2 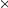 10^−16^ ***
	Residuals	4491	259904	57.87		

***The difference is greatly significant (P≪0.01).

a The data in African (ASW, LWK, MKK and YRI), East Asian (CHB, CHD and JPT) and European (CEU and TSI) are used for this analysis.

### Association between population groups and the genetic risk in the catabolic process

In this section, R (including R_p_, R_s_, and X) and Num (including Num_p, Num_s, and Num_x, which are the total number of mutations on population-shared, subpopulation-shared and group-specific SNPs respectively) were compared among groups statistically. Two statistical methods: ANOVA and student's t-test, were used. These two methods required approximate normality for the variable being analyzed. So first we validated the normality assumption. It was observed that R and Num were both distributed nearly normal (see Figure S6, S7, S8, S9, S10, S11, S12, S13, S14, S15, S16).

**Figure 2:**
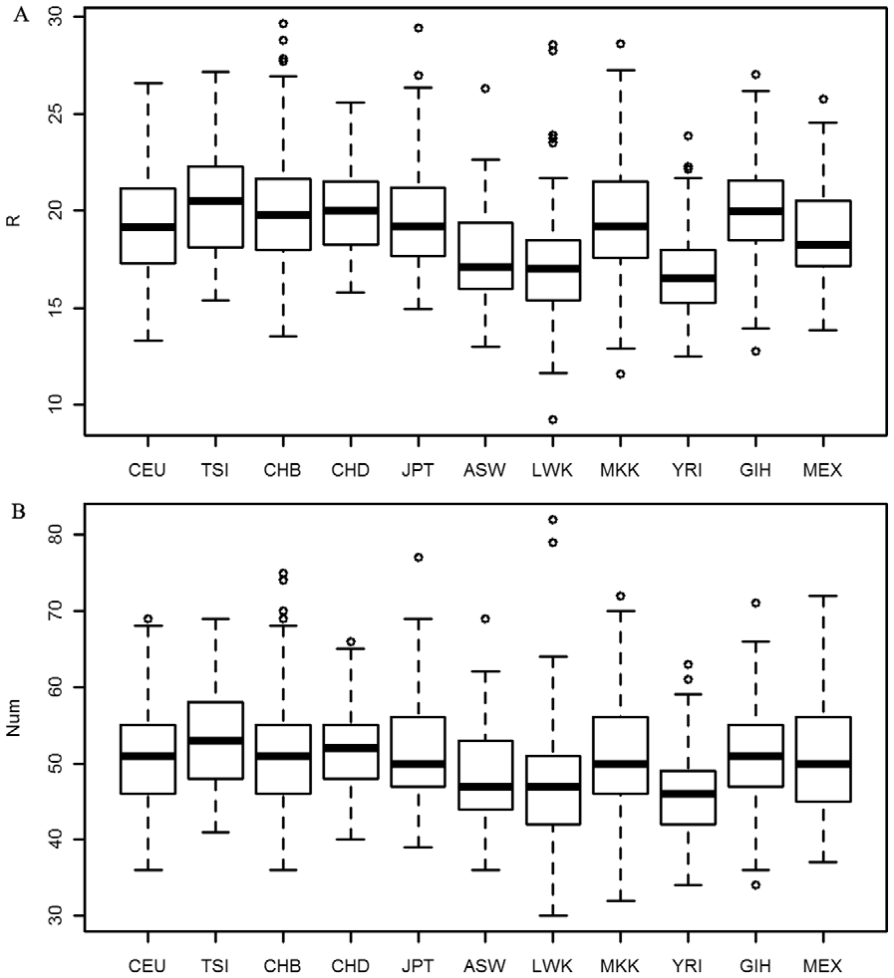
The overall genetic risk on all screened SNPs in catabolic process. R (subplot A) is the sum of harmful probabilities on all screened SNPs, and Num (subplot B) is the total number of mutations on all screened SNPs.

Our study revealed several features for R and Num in the catabolic process. First, the overall genetic risk was smaller in African groups than in other groups. R and Num among subpopulations and groups were significantly different with *P_Value*<0.01 (see Figure 2, Table 4 and Table S4 for details). R and Num in 3 of the 4 African groups (ASW, LWK and YRI) were significantly smaller than those in East Asian and European (P<0.01) with the exception of MKK whose R and Num were not smaller than East Asian and European (see Figure 2). Taking together the current results and the previous permutation results showing the bigger R and Num in background among Africans than other populations, we can suggest that the genetic risk during the catabolic process in most Africans (3 out of 4 groups) might be smaller than other populations. Lower genetic risk in the catabolic process might result in higher efficiency of generating energy, so these observations were also consistent with clinical observations of lower EE in Africans [2].

**Table 4:** Analysis of Variance for Observations (ANOVA) of genetic risk estimated by R on genes in catabolic process.^a^

	Df	Sum Sq.	Mean Sq.	F value	Pr(>F)
Gender	1	25.7	25.71	3.7328	0.05359
Subpopulation	2	1208.6	604.32	87.725	<2.2 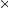 10^−16^ ***
Group	6	1006.7	167.78	24.354	<2.2 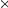 10^−16^ ***
Gender 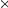 sub- population	2	20.6	10.32	1.4987	0.22385
Gender 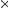 group	6	93.3	15.55	2.2579	0.03583*
Residuals	1192	8211.5	6.89		

***The difference is greatly significant (P≪0.01).

*The difference is significant (P<0.05).

a The data in African (ASW, LWK, MKK and YRI), East Asian (CHB, CHD and JPT) and European (CEU and TSI) are used for this analysis.

Previous clinical studies reported that EE in Asian was lower than in European [2]. In our study, R in TSI was shown to be significantly larger than JPT (P = 0.006264), but the significance was not observed between TSI and CHB or TSI and CHD (P = 0.07824 and 0.1095 respectively). However, our study did show that R in CEU was significantly smaller than CHB and CHD (P = 0.00776 and 0.003167), but was not significantly smaller than JPT (P = 0.1627). There was no significant difference of Num between Asian and European. Our study also reported that the significant difference of Num between males and females existed with *P_Value*<0.05 (see Table S4), but no significance of R was observed between males and females with *P_Value*>0.05 (see Table 4). Our study also tested the interaction between gender and group for R and Num. The results showed that R and Num in some groups were significant different between males and females with *P_Value*<0.05 (see Table 4 and S4 the details). Further analyses showed that in CHB and GIH, R and Num were significantly larger in males than in females (P<0.05), while in JPT and LWK, R and Num were significantly smaller in males than in females (P<0.05).

**Figure 3:**
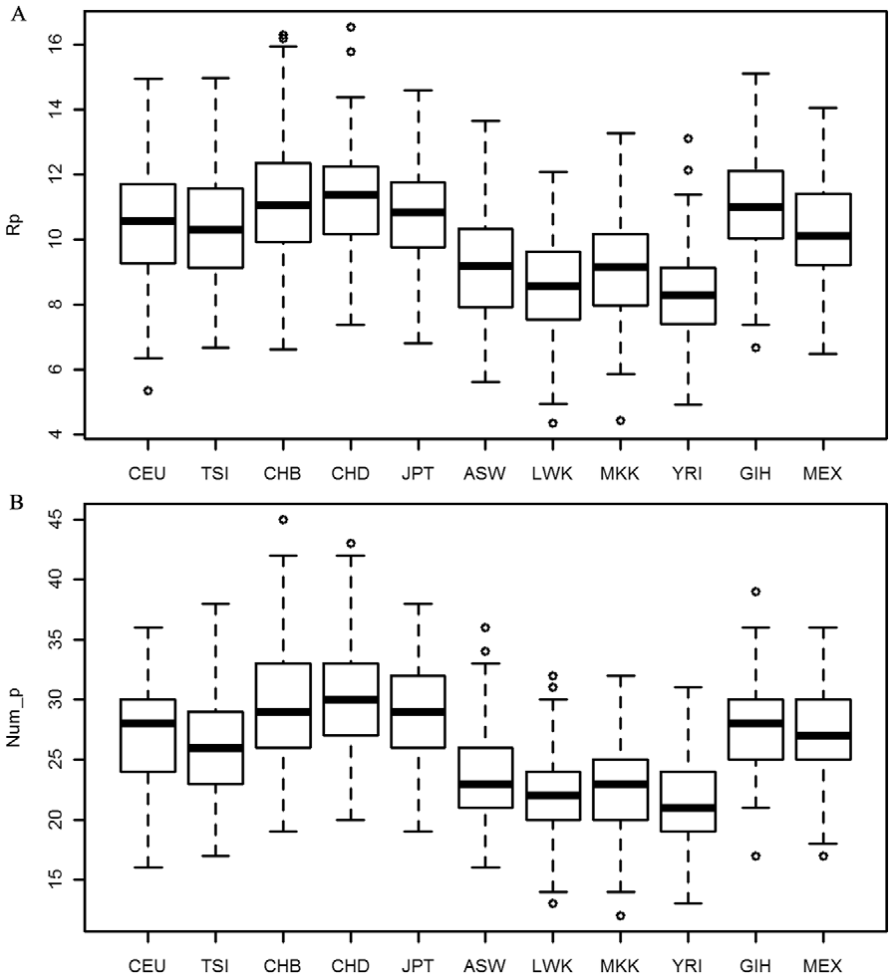
The genetic risk on population-shared SNPs in catabolic process. R_p_ (subplot A) is the sum of harmful probabilities on population-shared SNPs, and Num_p (subplot B) is the total number of mutations on population-shared SNPs.

Second, the genetic risk on population-shared SNPs in Africans were smaller than those in other groups. It was observed that R_p_ and Num_p within African subpopulations were very similar, but they were significantly smaller than those in non-African groups with *P-Value*<0.01 (t test, see Figure 3 for the details). As mentioned before, these mutations might be under selection. Therefore, this kind of risk was shared among human groups. Smaller genetic risk was consistently observed on these SNPs among Africans, which suggested that Africans might have lower genetic risk in the catabolic process naturally.

**Figure 4:**
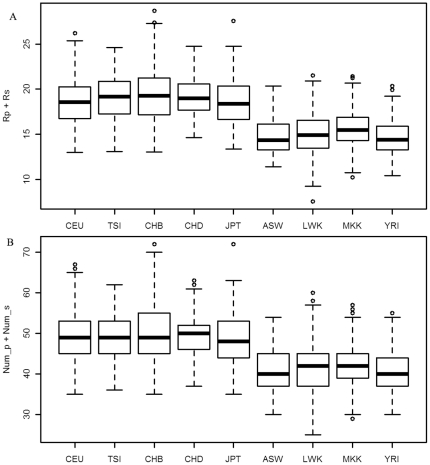
The genetic risk on population-shared and subpopulation-shared SNPs in catabolic process. R_p_+R_s_ (subplot A) is the sum of harmful probabilities on population-shared and subpopulation-shared SNPs, and Num_p+Num_s (subplot B) is the total number of mutations on population-shared SNPs. Genetic risk (R_p_+R_s_ in subplot A) and the sum of mutation number (Num_p+Num_s in subplot B) at population- and subpopulation-shared SNPs on candidate genes of coronary heart disease in human groups.

Third, the genetic risk in the catabolic process on population-shared and subpopulation-shared SNPs in African was smaller than other groups. Results of R_p_+R_s_ and Num_p+Num_s indicated that they were both significantly smaller in Africans than in East Asian and European with *P_Value<0.01* (t test, see Figure 4 for details).

**Figure 5:**
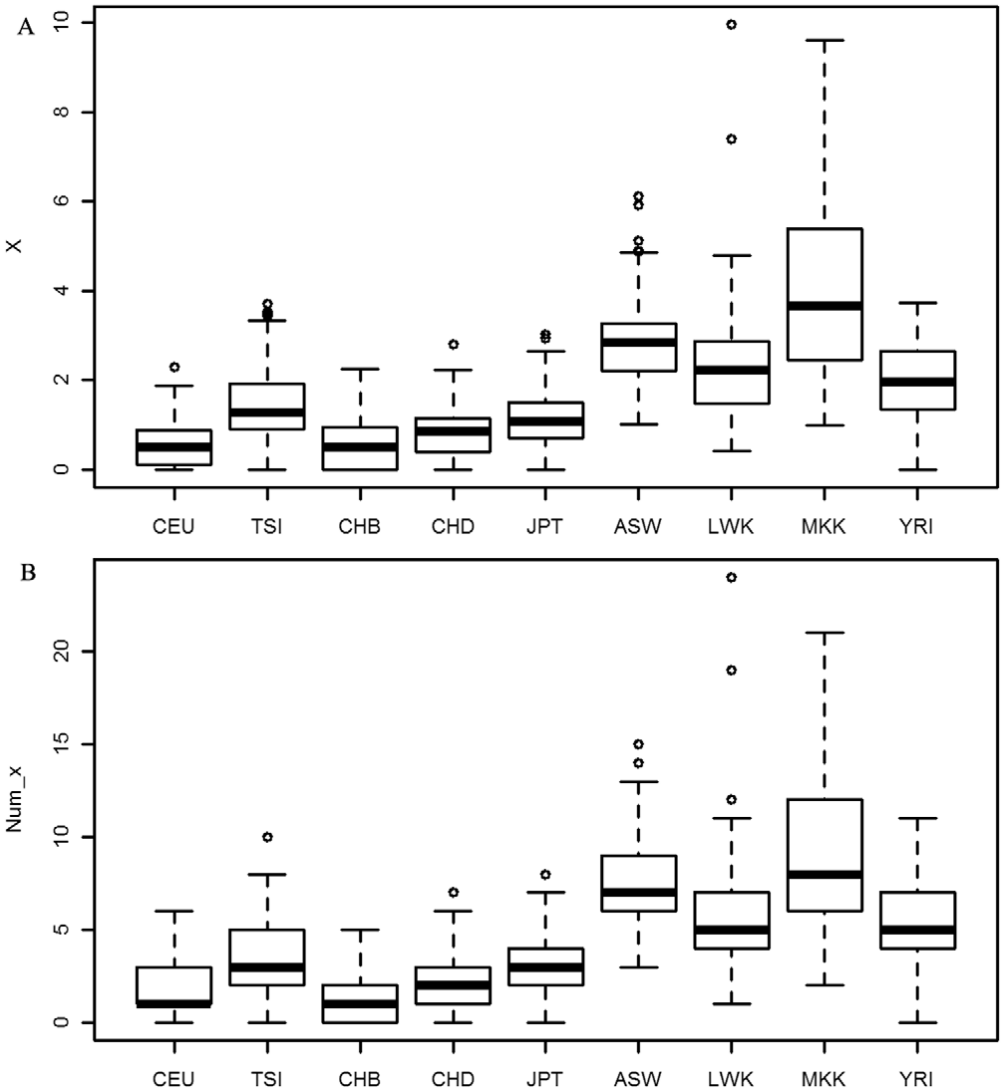
The genetic risk on group-specific SNPs in catabolic process. X (subplot A) is the sum of harmful probabilities on group-specific SNPs, and Num_x (subplot B) is the total number of mutations on group-specific SNPs.

Fourth, the genetic risk on group-specific SNPs in African was larger than those in East Asian and European. Both X and Num_x for CEU were the smallest among human groups (see Figure 5). From the permutation results described above after repeating 500 random samples from the whole human genome, it was also observed that the background genetic risk for group-specific SNPs in African is larger than those in East Asian and European (more detailed data not shown). This suggested that higher genetic risk for group-specific SNPs in the catabolic process could be likely a consequence of the higher background genetic risk in African for group-specific SNPs.

**Figure 6:**
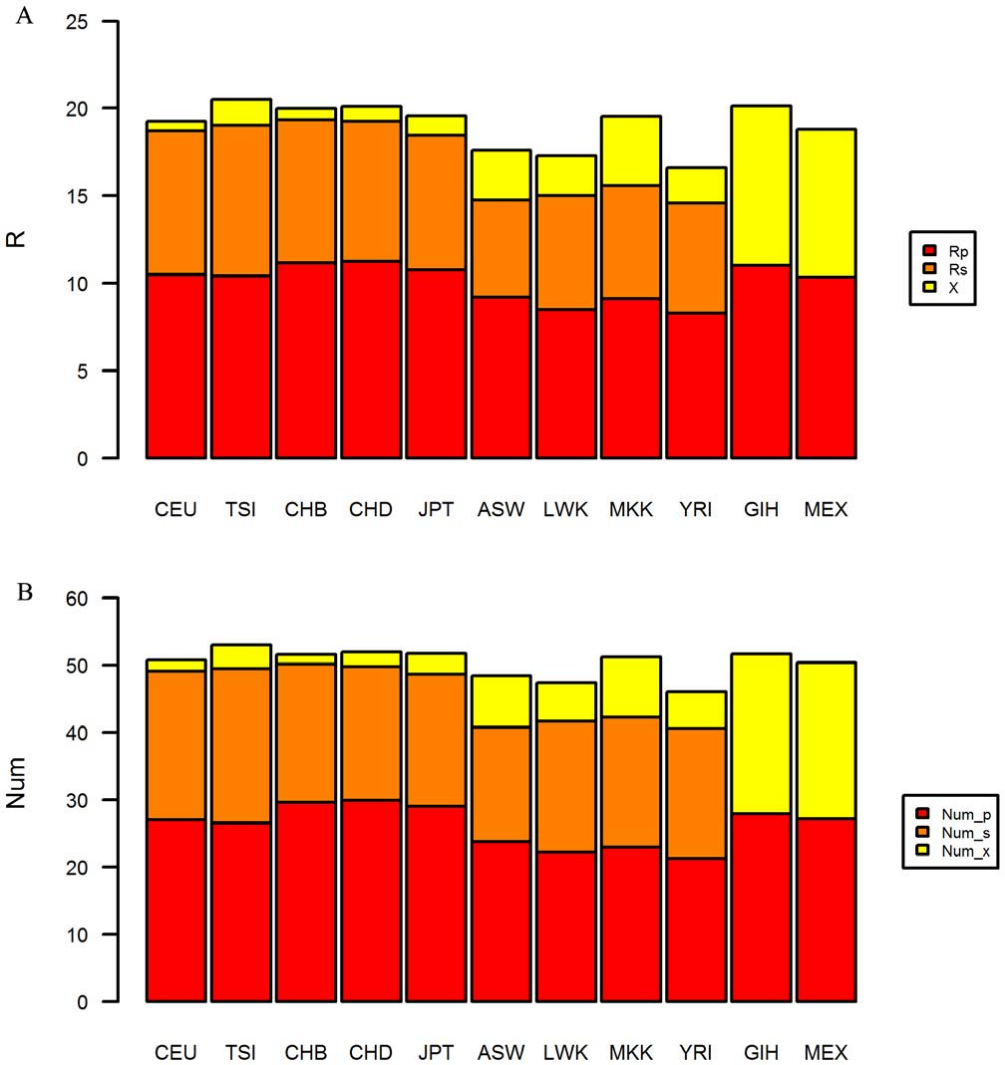
Proportions of components in mean genetic risk in human groups. In subplot A, R_p_, R_s_ and X (subplot A) are the sums of harmful probabilities on population-shared, subpopulation-shared and group-specific SNPs, respectively. In subplot B, Num_p, Num_s and Num_x are the total numbers of mutations on population-shared, subpopulation-shared and group-specific SNPs, respectively.

Fifth, most of the overall genetic risk came from population-shared and subpopulation-shared SNPs. The proportions of R_p_+R_s_ in R, or Num_p+Num_sin Num were larger than 80% (Figure 6), especially among Europeans and East Asians. R_p_+R_s_ were all lower in Africans than in Europeans and East Asians, which contributed largely to lower R in Africans (see Figure 2).

These observations suggested that in the catabolic process, 1. The genetic risk might be lower in most Africans than in Europeans and East Asians; 2. Most of genetic risk might originate from population- and subpopulation-shared SNPs; 3. The genetic risk on population- and subpopulation-shared SNPs was lower in Africans than in Europeans and East Asians, which contributed majorly to lower genetic risk in Africans.

## Discussion

In this study, many other genetic data were ignored in analyzing the genetic risk in the catabolic process, such as mutation data (SNP) in regulatory regions and frameshift mutation data, because they have not been well annotated. On the other hand, if these mutations are harmful enough, they may also result in the loss of function so that more missense mutations might be accumulated in coding region.

In our study, two indices – the total number of mutations (Num) and the sum of the harmful probability on each SNP (R) were considered in analyzing the genetic risk in catabolism. There were two assumptions when calculating these two indices. For Num, it was assumed that each screened SNP contributed equally to the genetic risk in catabolism. For R, it was assumed that each SNP contributed unequally. At this point, R should be more realistic and accurate as a proxy of the genetic risk than Num. However, based on the observations of R and Num in our study, almost the same results and inferences were obtained (see ure 2–5 and Figure S6, S7, S8, S9, S10, S11, S12, S13, S14, S15, S16).

In this study, it was observed that every individual sampled had more than 1 mutation at 231 SNPs in the catabolic process, and mean number of mutation was 48.37. Although so many detrimental mutations occur in our genomes, why are most of us still healthy? The possible reasons might be: First, most mutations are recessive, so the harmful effect is shielded. Second, in human genomes there are many duplicate genes [11]–[14]. As a gene becomes nonfunctional because of mutations, this gene's duplicate genes might pick up the job. Third, if a pathway is broken down because of the loss of some essential functions on genes cause by mutations, other pathways in biological complicated networks may also take over the role of the breakdown pathway [11], [14]. More mutations in genomes mean more genetic risk in the catabolic process.

Asians have also been shown to have lower EE than Europeans [2]. In this study, it was observed that TSI had larger genetic risk in the catabolic processes than JPT, while CEU had smaller genetic risk than CHB and CHD (see above and Figure 2). Our observations indicated that the difference of the genetic risk within Asian and European was so apparent that results between Asian and European were heterogeneous.

As observed above, Num and R in most Africans (ASW, LWK and YRI) were significantly smaller than those in European and East Asian. It was reported that if a gene lost its function due to accumulated mutations, the duplicate gene might take over its job, but the efficiency of doing the same work might be decreased [11]. For European, more genetic risk in the catabolic process may cause the decrease of efficiency in generating energy, so it has been observed that Europeans need more EE than Africans in the same routine activity. In the same way, the increase of efficiency in generating energy might contribute to more powerful capability in African-descendant athletes during athletic competitions.

Previous studies reported that obesity and overweight were more common in African-American than in European-American [8], which also might be explained by our results. Due to lower genetic risk in the catabolic process in Africans, the higher-level efficiency in generating energy might result in consuming smaller volume of body mass (for example fat) in African-Americans than in European-Americans when doing the same routine activities. If an African-American wants to consume the same volume of body mass, he (or she) needs to do more activities. However, in reality the total amounts of routine activities between African- and European-Americans are similar under the same or similar environments, so African-Americans are more likely to be overweight or obese. Therefore, the smaller genetic risk in the catabolic process might be at the core of understanding lower EE, more athletic capability and higher prevalence of obesity in Africans.

## Methods

### Genotype data

Genotype data used in this study was downloaded from HapMap data (Hapmap Public Release #3 on May 28, 2010, http://hapmap.ncbi.nlm.nih.gov/). The list of genes in the catabolic process were downloaded from Gene Ontology (GO: 0009056) (http://www.geneontology.org), and the total number of genes involved in the catabolic process was 1601 in human genome. SNPs with missense mutations on these genes were downloaded from NCBI dbSNP database (ftp://ftp.ncbi.nih.gov/snp/). The harmful probability for each SNP estimated with Polyphen-2 was downloaded from http://genetics.bwh.harvard.edu/pph2/dbsearch.shtml. For a false positive rate of 20%, in PolyPhen-2 the true positive prediction rate was 92%, trained on HumDiv dataset [10]. So the HumDiv-trained score of harmful effects for mutation was referenced.

### Imputation

There were some missing genotype data in Hapmap data, so hapmap genotype data was imputed with 1000 Genome reference panels (http://www.sph.umich.edu/csg/abecasis/MACH/download/1000G-2010-08.html). MACH was the software used for imputation and was downloaded from http://www.sph.umich.edu/csg/abecasis/MACH/download/. During imputation, when a given proportion (0.02) of known genotype data were masked for test, the error rate per genotype was 0.0267±0.0041 (mean±SD), and the error rate per allele was 0.0141±0.0022.

### Calculation of indices for the genetic risk

Two parameters (R and Num) for the genetic risk in the catabolic process were calculated. With the assumption that the harmful effect of each mutation was equal, we calculated the total number of mutations at all screened SNPs (Num) as an index for the genetic risk of mutations in catabolic process. Additionally, with the assumption that the harmful effect was unequal for each SNP, we also calculated the sum of the harmful effect of each screened SNPs (R) as another index of the genetic risk. Let R_i_ be the genetic risk for the i-th SNP. When at this SNP site the genotype was wild-type (or major frequency allele) homozygous, R_i_ = 0; when the genotype was heterozygous, if mutant allele was dominant (described in OMIM database, Online Mendelian Inheritance in Man), R_i_ = r_i_ (r_i_ is the harmful probability estimated by Polyphen-2), otherwise R_i_ = r_i_/2; when the genotype was mutant homozygous, R_i_ = r_i_. Then R on an individual was calculated by
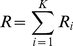
(2)Where K is the number of SNPs screened.

In Hapmap genotype data some SNPs were shared in all human groups (or human population), and some were shared in subpopulations. The set of SNPs shared among human population were termed population-shared SNPs, those shared in human subpopulations were termed subpopulation-shared SNPs, and those not shared in population and subpopulations were termed group-specific SNPs. Thus the genetic risk in catabolic process at the population level was divided into three parts.
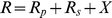
(3)In which R is the total harmful effect (or genetic risk) at the population level, R_p_, R_s_ and X are the risks of mutations on population- and subpopulation-shared SNPs, and group-specific SNPs, respectively.

### Permutation test

The list of genes in human genome was obtained from http://www.geneontology.org, and 18161 genes in total were downloaded. Of these 18161 genes, 1601 (the number of genes involved in the catabolic process) genes were re-sampled randomly up to 500 times, followed by the calculations of R and Num for these genes. Additionally, we also performed another permutation test to re-sample randomly 231 SNPs (the number of SNPs screened from the catabolic process) from 3357 common harmful SNPs screened on 18161 human genes in human genomes. And this process of re-sampling also repeats 500 times. At each round of re-sampling process, mean R and mean Num were calculated for each human group. And then the summary of mean R and mean Num for 500 repetitions we shown in Figure S4 and S5. The results in these two permutation tests were treated as an estimation of the background noise level, and then were used for adjustment when analyzing the targeted marker set.

### Statistical methods

Unpaired two-tailed Student's test and Analysis of Variance for Observations (ANOVA) test were performed to compare results between and among groups.

## Supporting Information

Figure S1 **Patterns for MAFs on population-shared SNPs in catabolic process in Africans and Asian.** Results are for ASW (African ancestry in Southwest USA), LWK (Luhya in Webuye, Kenya), CHB (Han Chinese in Beijing, China), and JPT (Japanese in Tokyo, Japan).
(TIF)

Figure S2 **Patterns for MAFs on population-shared SNPs in catabolic process in European and other groups.** Results are for CEU (Utah residents with Northern and Western European ancestry from the CEPH collection), and TSI (Toscans in Italy), GIH (Gujarati Indians in Houston, Texas) and MEX(Mexican ancestry in Los Angeles, California).
(TIF)

Figure S3 **Patterns for MAFs (minor allele frequency) of population-shared SNPs on screened genes in catabolism process in MKK (Maasai in Kinyawa, Kenya), YRI (Yoruba in Ibadan, Nigeria), and CHD (Chinese in Metropolitan Denver, Colorado).**
(TIF)

Figure S4 **The genetic risks (mean R and mean Num) on genes re-sampled randomly 1601 genes from 18161 genes in human genomes.** And this process of re-sampling 1601 genes from 18161 human genes repeats 500 times. Mean R (subplot A) are means of the sum of harmful probabilities at screened SNPs on re-sampled genes, and mean Num (subplot B) are means of the total number of mutations at screened SNPs on re-sampled genes for 500 permutation repetitions.
(TIF)

Figure S5 **The genetic risks (mean R and mean Num) on 231 SNPs (the number of SNPs screened from the catabolic process) re-sampled randomly from 3357 SNPs screened on 18161 human genes in human genomes.** And this process of re-sampling repeats 500 times. Mean R (subplot A) are means of the sum of harmful probabilities on 231 re-sampled SNPs, and mean Num (subplot B) are means of the total number of mutations on 231 re-sampled SNPs for 500 permutation repetitions.
(TIF)

Figure S6 **Distributions of genetic risk (R in subplot A) and the sum of mutations (Num in subplot B) at all screened SNPs on genes of catabolism process in ASW (African ancestry in Southwest USA).**
(TIF)

Figure S7 **Distributions of genetic risk (R in subplot A) and the sum of mutations (Num in subplot B) at all screened SNPs on genes of catabolism process in CEU (Utah residents with Northern and Western European ancestry from the CEPH collection).**
(TIF)

Figure S8 **Distributions of genetic risk (R in subplot A) and the sum of mutations (Num in subplot B) at all screened SNPs on genes of catabolism process in CHB (Han Chinese in Beijing, China).**
(TIF)

Figure S9 **Distributions of genetic risk (R in subplot A) and the sum of mutations (Num in subplot B) at all screened SNPs on genes of catabolism process in CHD (Chinese in Metropolitan Denver, Colorado).**
(TIF)

Figure S10 **Distributions of genetic risk (R in subplot A) and the sum of mutations (Num in subplot B) at all screened SNPs on genes of catabolism process in GIH (Gujarati Indians in Houston, Texas).**
(TIF)

Figure S11 **Distributions of genetic risk (R in subplot A) and the sum of mutations (Num in subplot B) at all screened SNPs on genes of catabolism process in JPT (Japanese in Tokyo, Japan).**
(TIF)

Figure S12 **Distributions of genetic risk (R in subplot A) and the sum of mutations (Num in subplot B) at all screened SNPs on genes of catabolism process in LWK (Luhya in Webuye, Kenya).**
(TIF)

Figure S13 **Distributions of genetic risk (R in subplot A) and the sum of mutations (Num in subplot B) at all screened SNPs on genes of catabolism process in MEX (Mexican ancestry in Los Angeles, California).**
(TIF)

Figure S14 **Distributions of genetic risk (R in subplot A) and the sum of mutations (Num in subplot B) at all screened SNPs on genes of catabolism process in MKK (Maasai in Kinyawa, Kenya).**
(TIF)

Figure S15 **Distributions of genetic risk (R in subplot A) and the sum of mutations (Num in subplot B) at all screened SNPs on genes of catabolism process in TSI (Toscans in Italy).**
(TIF)

Figure S16 **Distributions of genetic risk (R in subplot A) and the sum of mutations (Num in subplot B) at all screened SNPs on genes of catabolism process in YRI (Yoruba in Ibadan, Nigeria).**
(TIF)

Table S1 **182 candidate genes in catabolism process with possible harmful variants.**
(DOC)

Table S2 **231 common (MAF>0.05) SNPs screened with possible harmful missense mutations (r_i_>0.2) on genes in catabolism process.**
(DOC)

Table S3 **Minor allele frequencies (MAFs) for 231 common (MAF>0.05) SNPs screened with possibly harmful missense mutations (r_i_>0.2) on genes in catabolism process.**
(DOC)

Table S4 **Analysis of Variance for Observations (ANOVA) of Num in human groups.**
(DOC)
