# Supplementary figures and images for: Smaller Genetic Risk in Catabolic Process Explains Lower Energy Expenditure, More Athletic Capability and Higher Prevalence of Obesity in Africans
Source: PLoS One. 2011 Oct 10;6(10):e26027. doi: 10.1371/journal.pone.0026027 (PMC3189926; doi:10.1371/journal.pone.0026027)

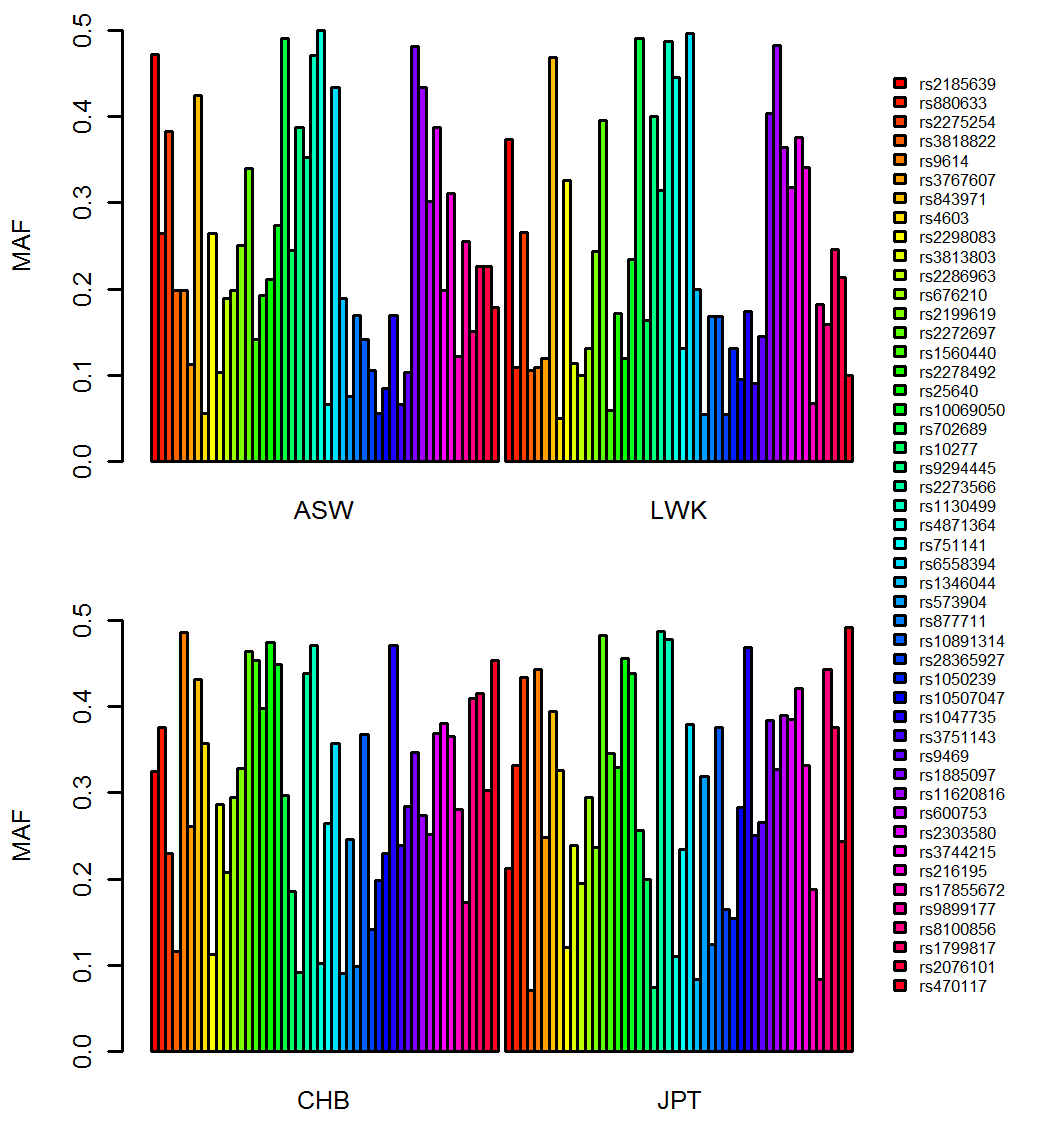

Supplement: Figure S1 — Patterns for MAFs on population-shared SNPs in catabolic process in Africans and Asian. Results are for ASW (African ancestry in Southwest USA), LWK (Luhya in Webuye, Kenya), CHB (Han Chinese in Beijing, China), and JPT (Japanese in Tokyo, Japan). (TIF) [file pone.0026027.s001.tif]

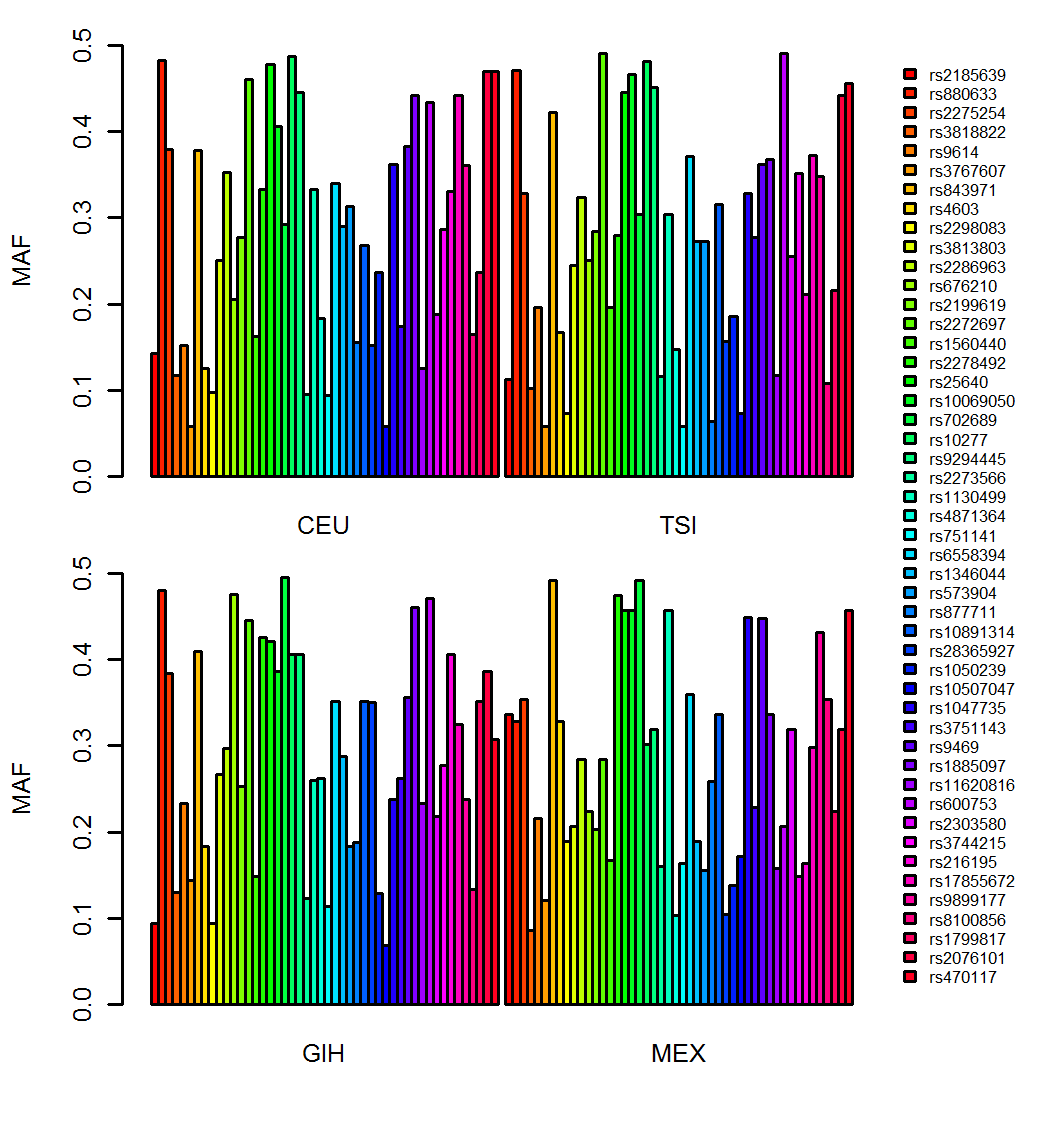

Supplement: Figure S2 — Patterns for MAFs on population-shared SNPs in catabolic process in European and other groups. Results are for CEU (Utah residents with Northern and Western European ancestry from the CEPH collection), and TSI (Toscans in Italy), GIH (Gujarati Indians in Houston, Texas) and MEX(Mexican ancestry in Los Angeles, California). (TIF) [file pone.0026027.s002.tif]

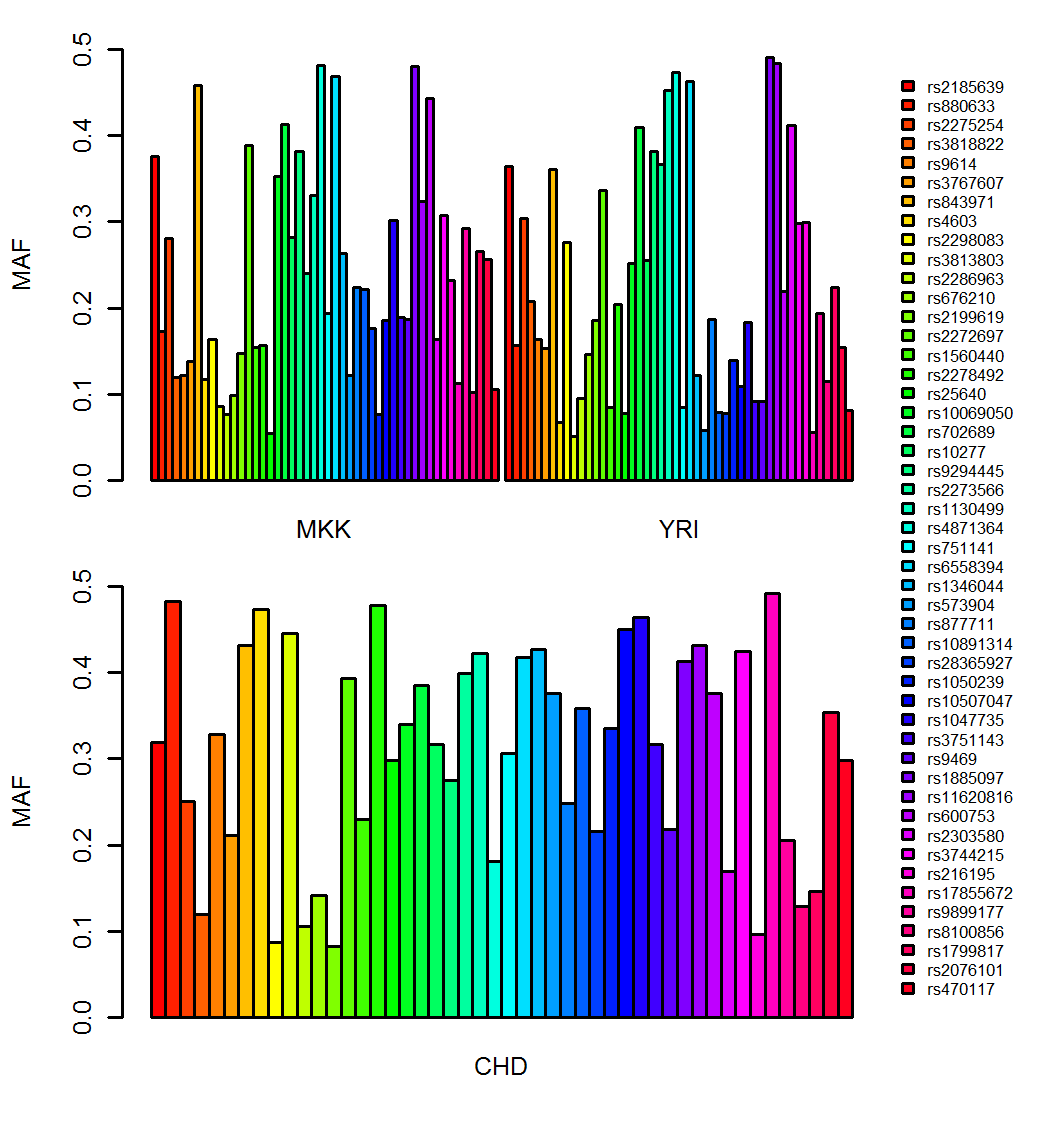

Supplement: Figure S3 — Patterns for MAFs (minor allele frequency) of population-shared SNPs on screened genes in catabolism process in MKK (Maasai in Kinyawa, Kenya), YRI (Yoruba in Ibadan, Nigeria), and CHD (Chinese in Metropolitan Denver, Colorado). (TIF) [file pone.0026027.s003.tif]

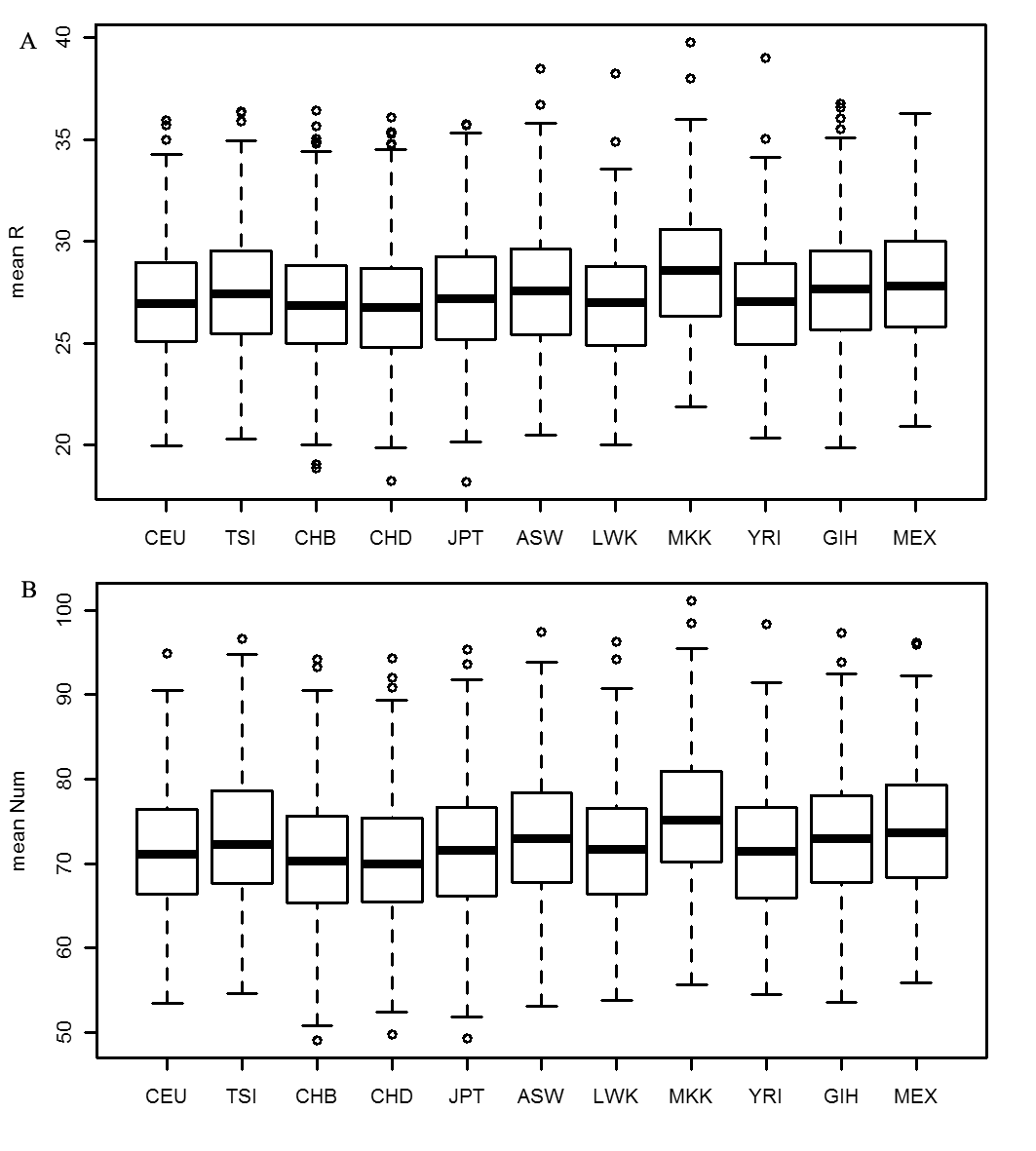

Supplement: Figure S4 — The genetic risks (mean R and mean Num) on genes re-sampled randomly 1601 genes from 18161 genes in human genomes. And this process of re-sampling 1601 genes from 18161 human genes repeats 500 times. Mean R (subplot A) are means of the sum of harmful probabilities at screened SNPs on re-sampled genes, and mean Num (subplot B) are means of the total number of mutations at screened SNPs on re-sampled genes for 500 permutation repetitions. (TIF) [file pone.0026027.s004.tif]

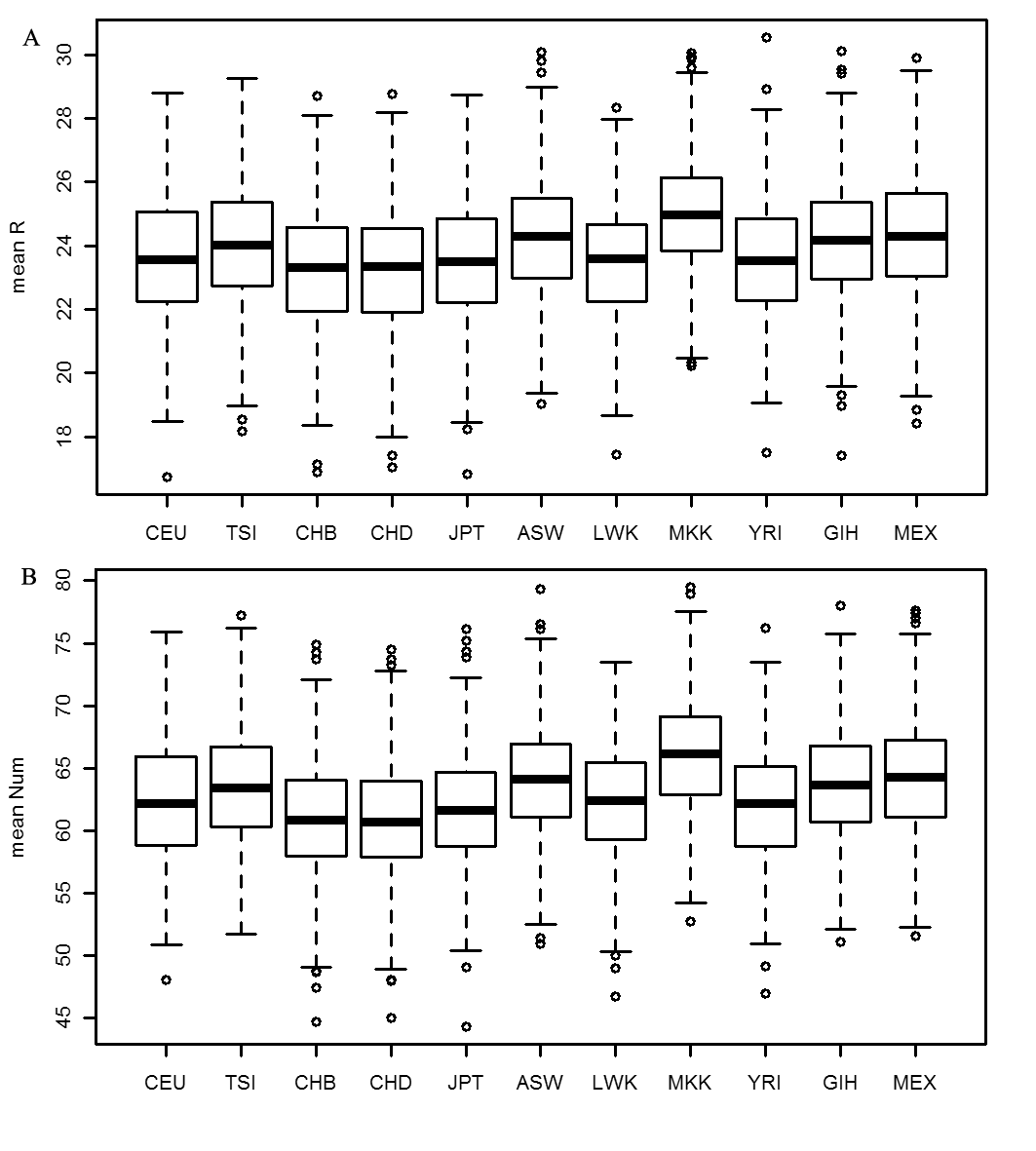

Supplement: Figure S5 — The genetic risks (mean R and mean Num) on 231 SNPs (the number of SNPs screened from the catabolic process) re-sampled randomly from 3357 SNPs screened on 18161 human genes in human genomes. And this process of re-sampling repeats 500 times. Mean R (subplot A) are means of the sum of harmful probabilities on 231 re-sampled SNPs, and mean Num (subplot B) are means of the total number of mutations on 231 re-sampled SNPs for 500 permutation repetitions. (TIF) [file pone.0026027.s005.tif]

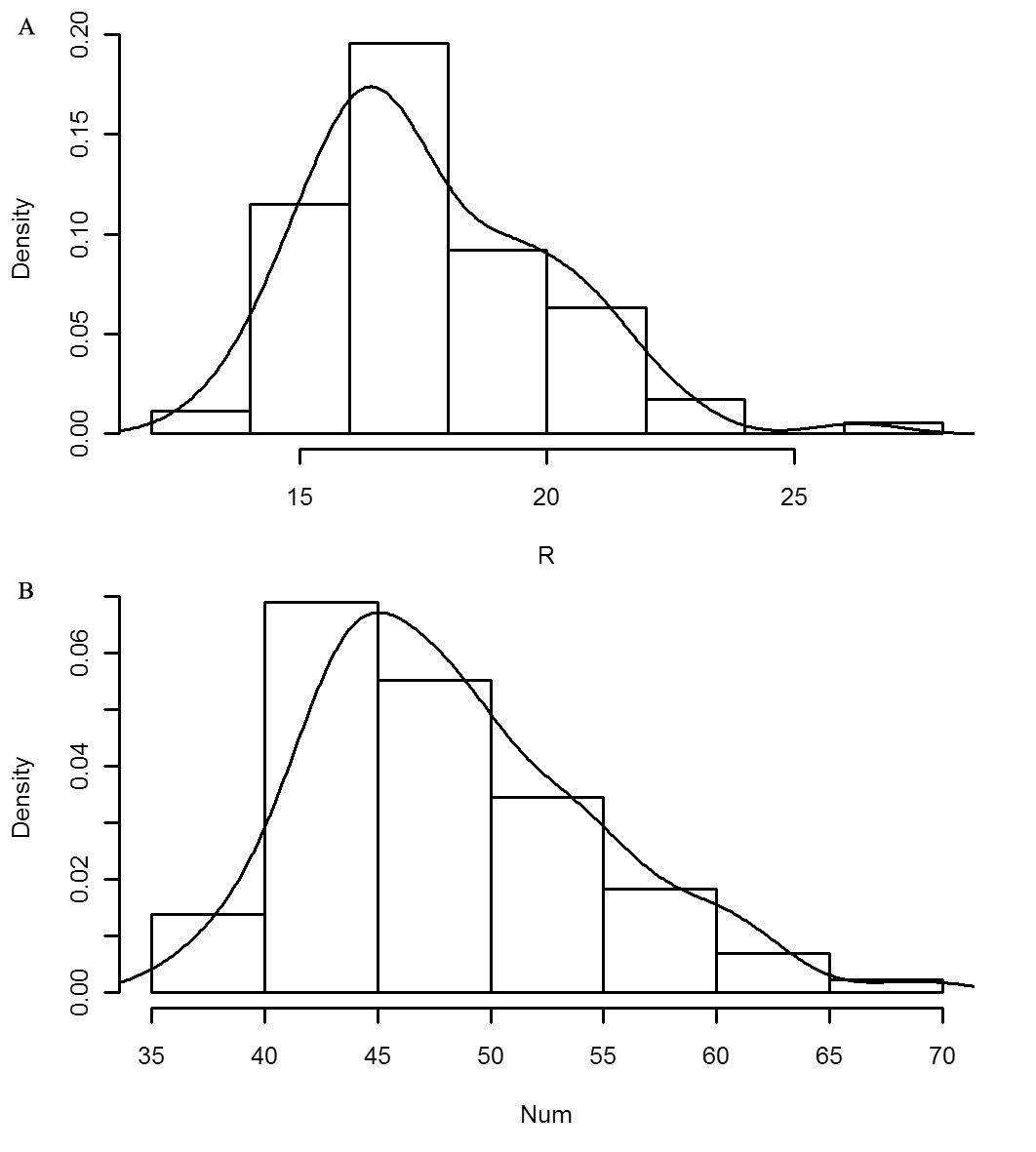

Supplement: Figure S6 — Distributions of genetic risk (R in subplot A) and the sum of mutations (Num in subplot B) at all screened SNPs on genes of catabolism process in ASW (African ancestry in Southwest USA). (TIF) [file pone.0026027.s006.tif]

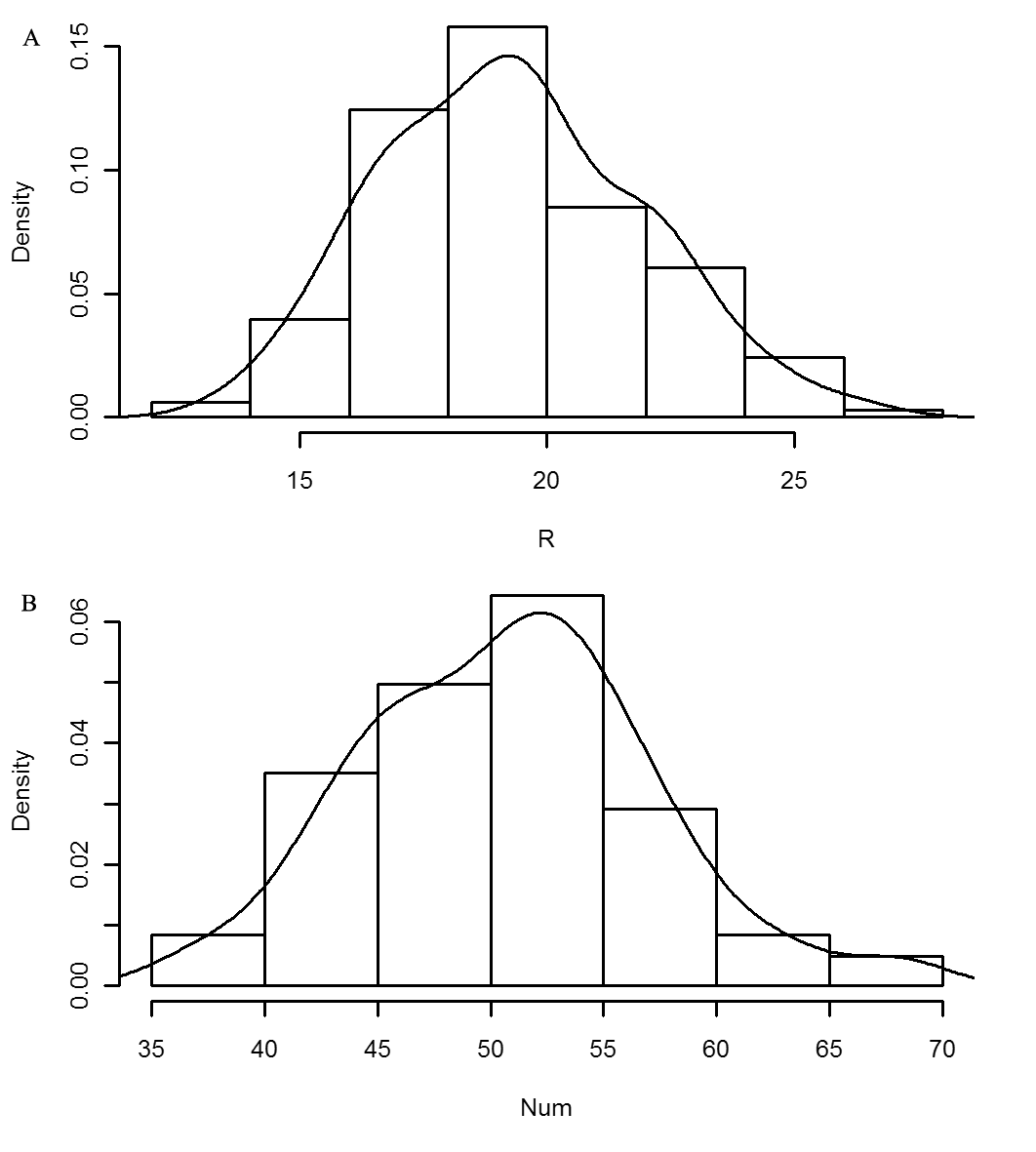

Supplement: Figure S7 — Distributions of genetic risk (R in subplot A) and the sum of mutations (Num in subplot B) at all screened SNPs on genes of catabolism process in CEU (Utah residents with Northern and Western European ancestry from the CEPH collection). (TIF) [file pone.0026027.s007.tif]

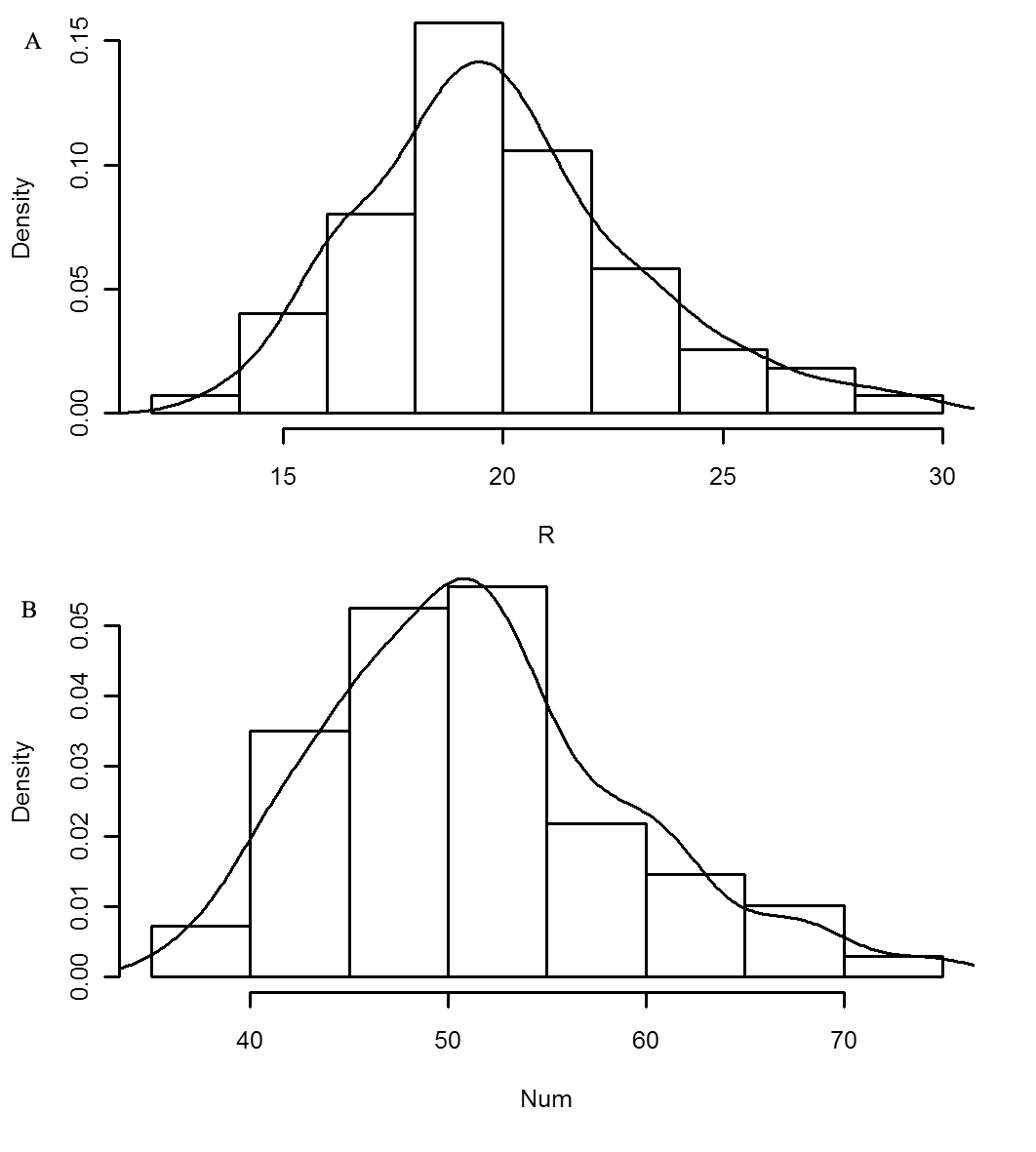

Supplement: Figure S8 — Distributions of genetic risk (R in subplot A) and the sum of mutations (Num in subplot B) at all screened SNPs on genes of catabolism process in CHB (Han Chinese in Beijing, China). (TIF) [file pone.0026027.s008.tif]

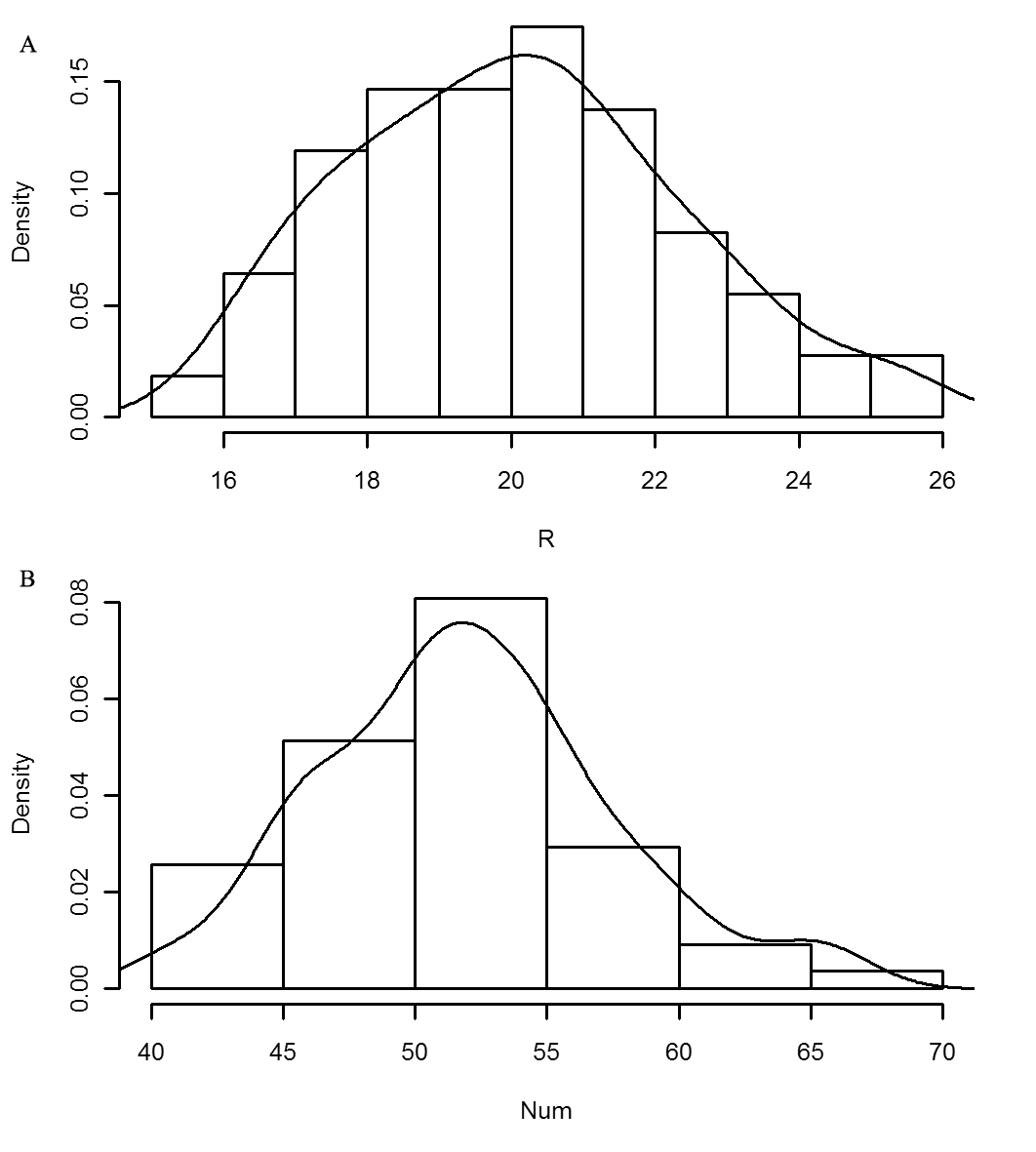

Supplement: Figure S9 — Distributions of genetic risk (R in subplot A) and the sum of mutations (Num in subplot B) at all screened SNPs on genes of catabolism process in CHD (Chinese in Metropolitan Denver, Colorado). (TIF) [file pone.0026027.s009.tif]

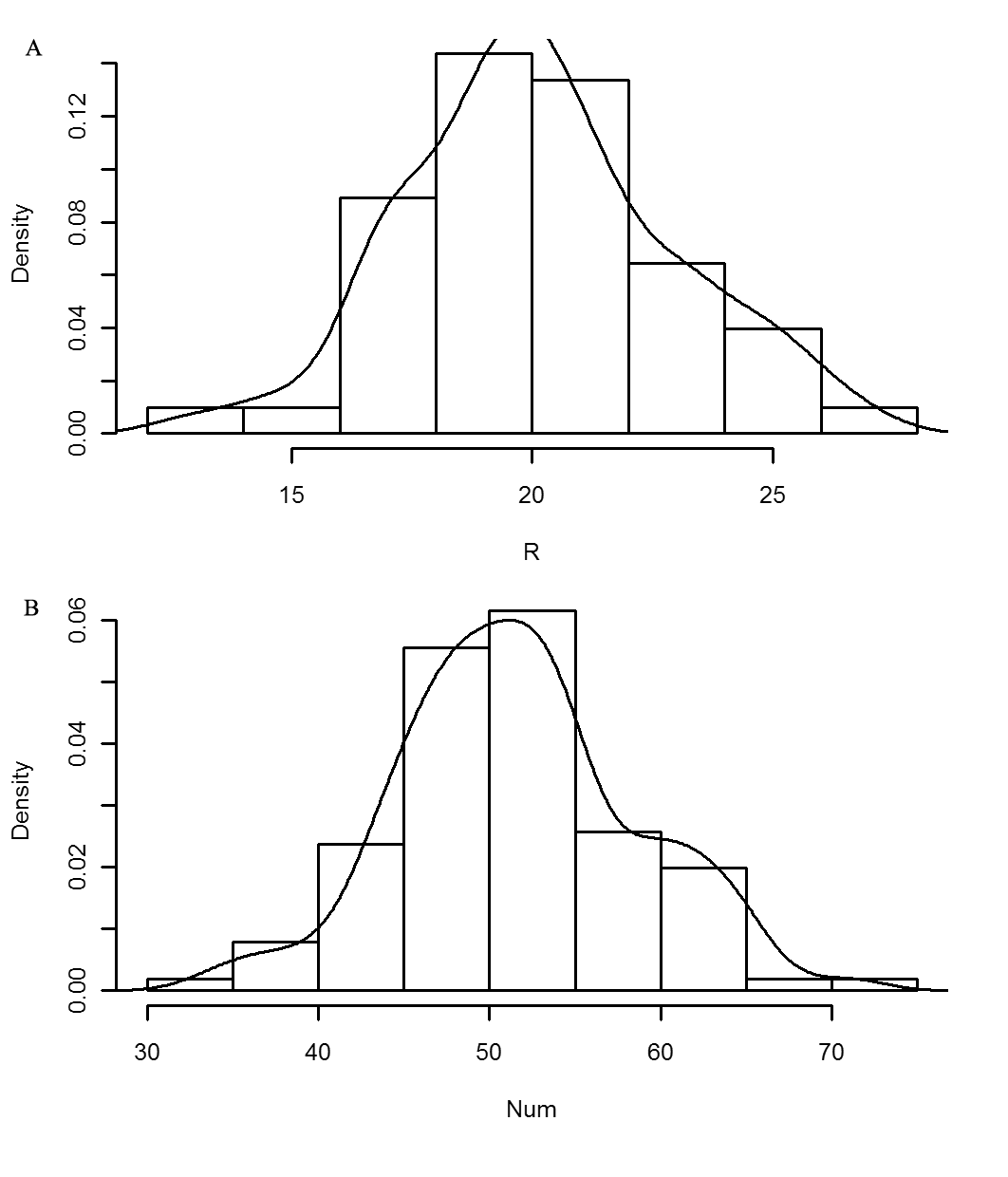

Supplement: Figure S10 — Distributions of genetic risk (R in subplot A) and the sum of mutations (Num in subplot B) at all screened SNPs on genes of catabolism process in GIH (Gujarati Indians in Houston, Texas). (TIF) [file pone.0026027.s010.tif]

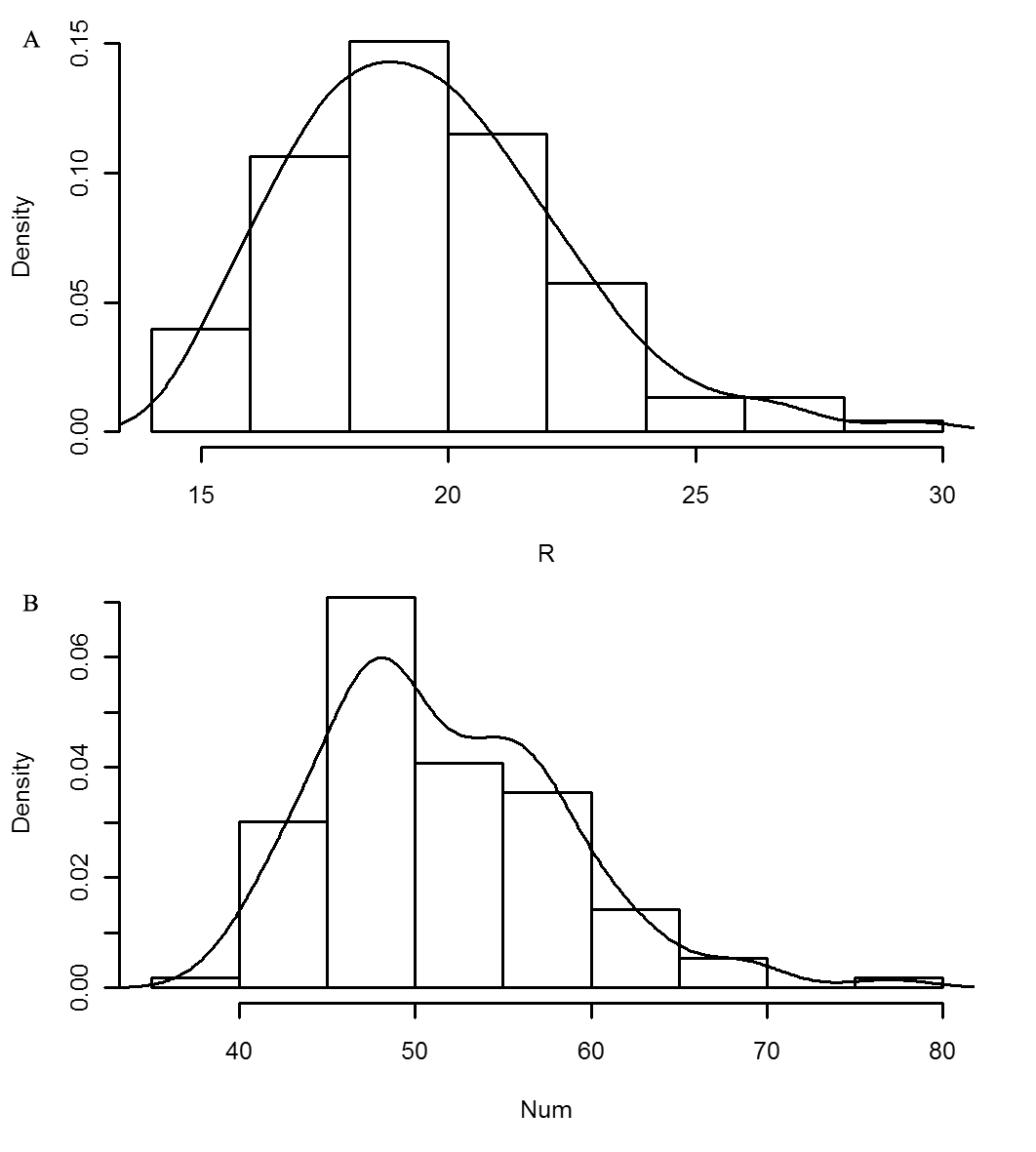

Supplement: Figure S11 — Distributions of genetic risk (R in subplot A) and the sum of mutations (Num in subplot B) at all screened SNPs on genes of catabolism process in JPT (Japanese in Tokyo, Japan). (TIF) [file pone.0026027.s011.tif]

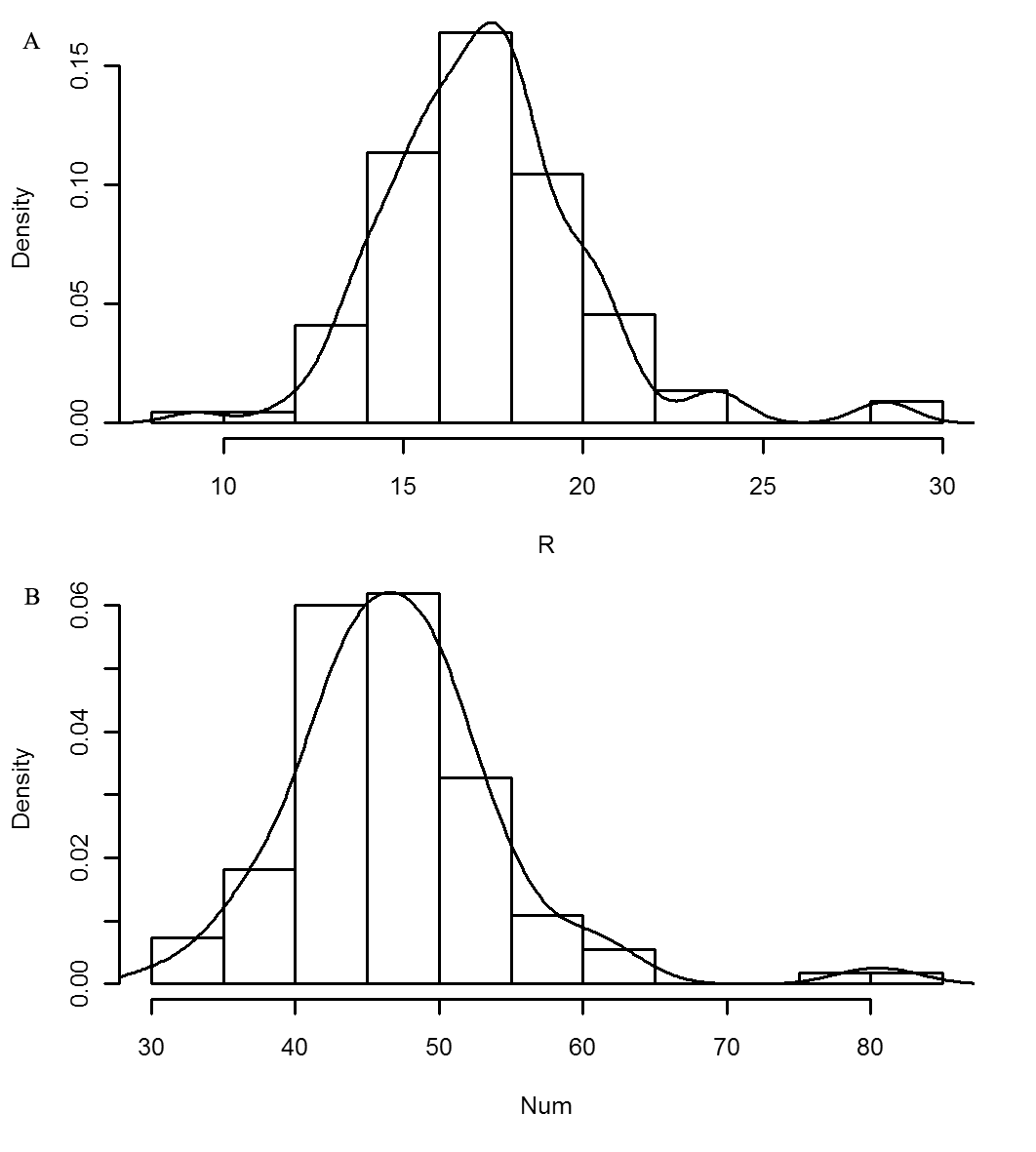

Supplement: Figure S12 — Distributions of genetic risk (R in subplot A) and the sum of mutations (Num in subplot B) at all screened SNPs on genes of catabolism process in LWK (Luhya in Webuye, Kenya). (TIF) [file pone.0026027.s012.tif]

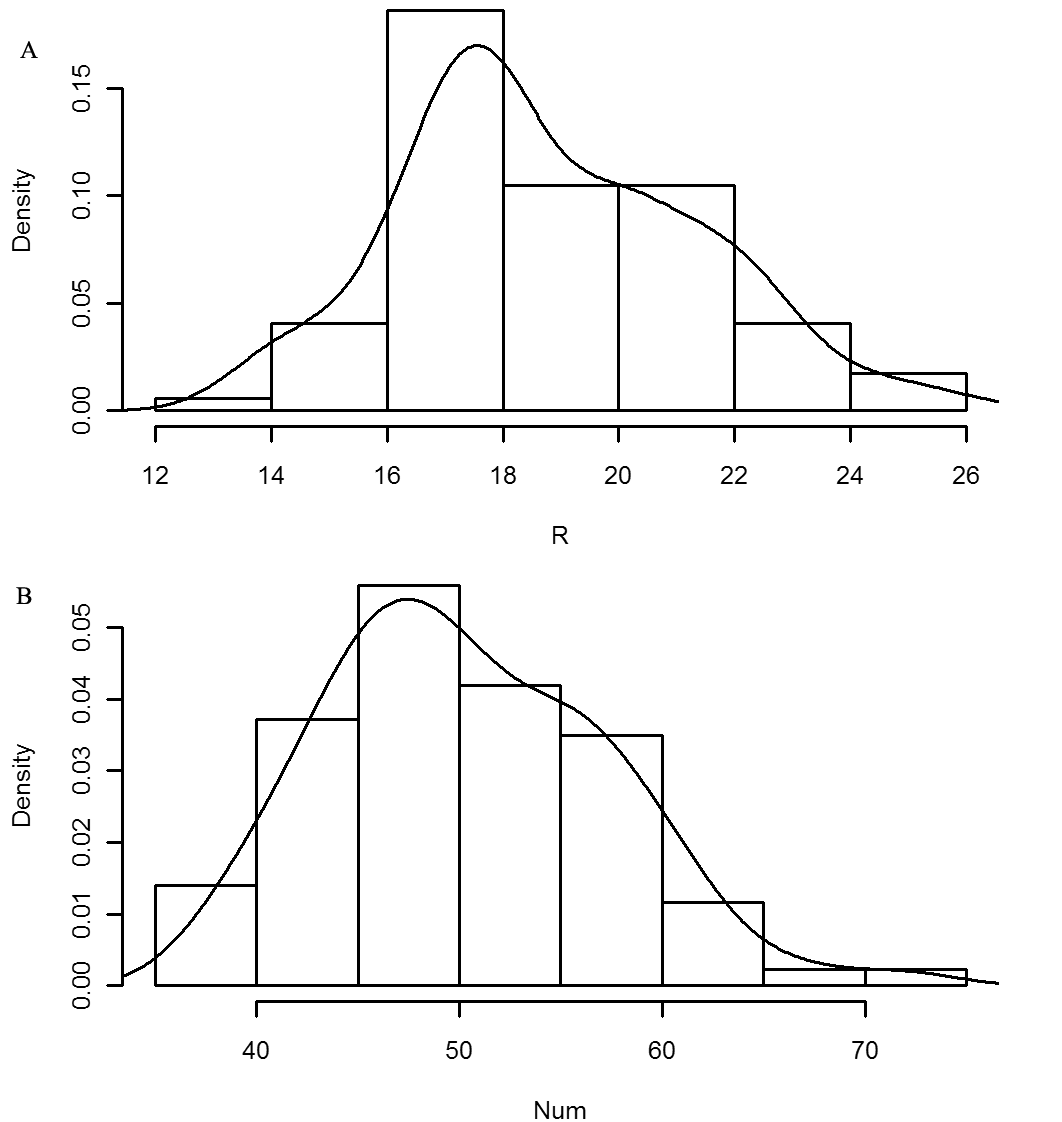

Supplement: Figure S13 — Distributions of genetic risk (R in subplot A) and the sum of mutations (Num in subplot B) at all screened SNPs on genes of catabolism process in MEX (Mexican ancestry in Los Angeles, California). (TIF) [file pone.0026027.s013.tif]

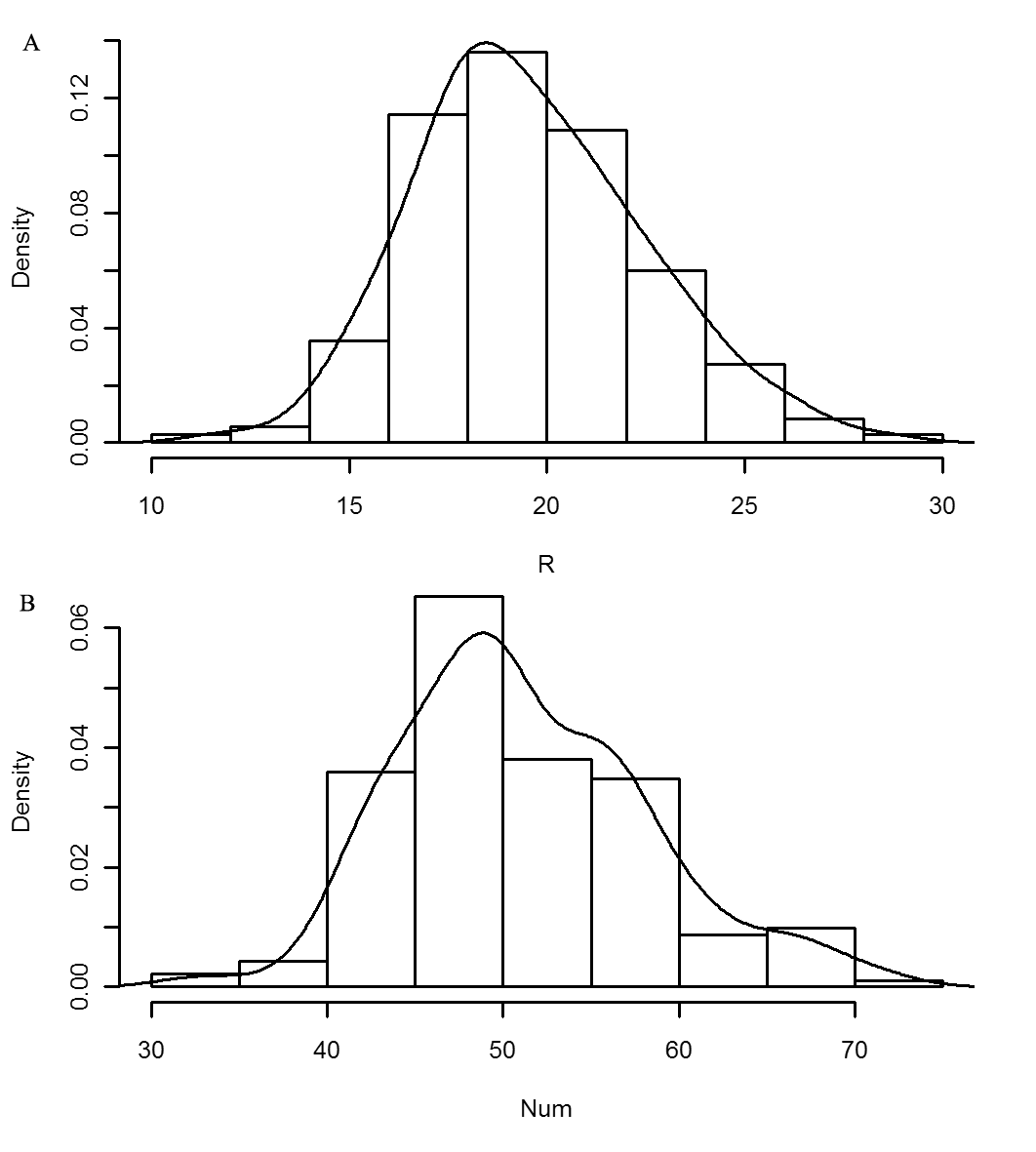

Supplement: Figure S14 — Distributions of genetic risk (R in subplot A) and the sum of mutations (Num in subplot B) at all screened SNPs on genes of catabolism process in MKK (Maasai in Kinyawa, Kenya). (TIF) [file pone.0026027.s014.tif]

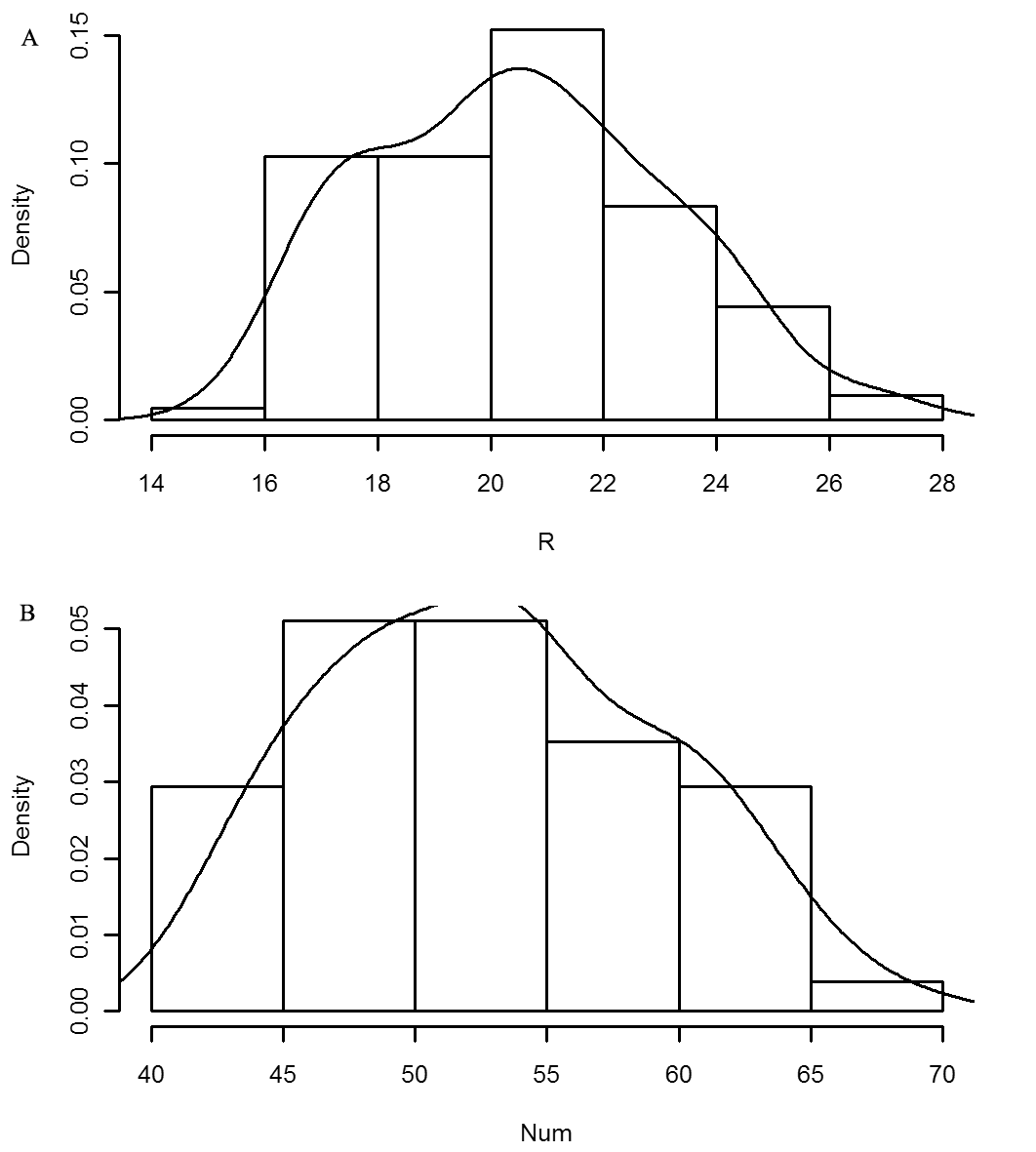

Supplement: Figure S15 — Distributions of genetic risk (R in subplot A) and the sum of mutations (Num in subplot B) at all screened SNPs on genes of catabolism process in TSI (Toscans in Italy). (TIF) [file pone.0026027.s015.tif]

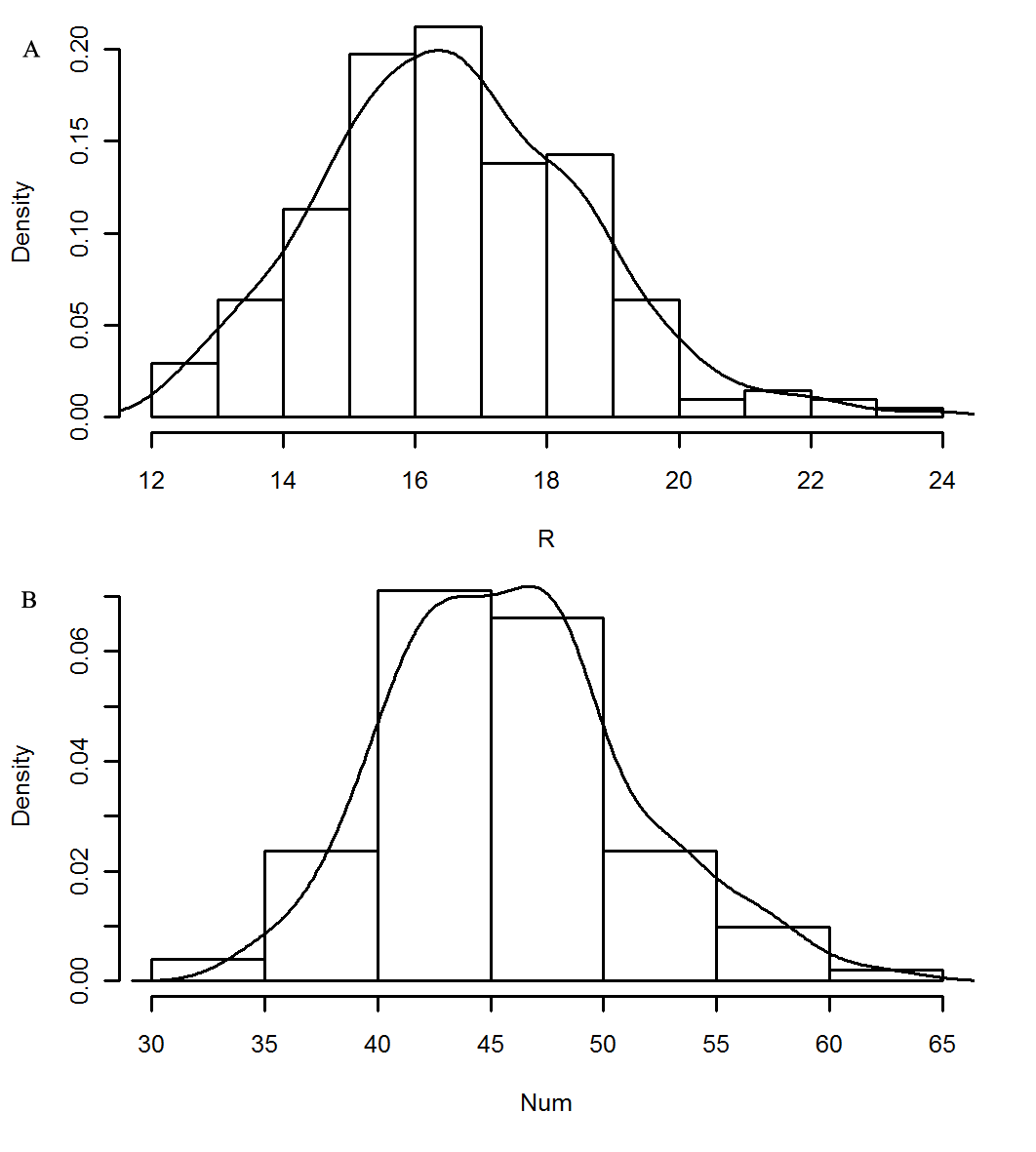

Supplement: Figure S16 — Distributions of genetic risk (R in subplot A) and the sum of mutations (Num in subplot B) at all screened SNPs on genes of catabolism process in YRI (Yoruba in Ibadan, Nigeria). (TIF) [file pone.0026027.s016.tif]
