# Supplementary material for: Smaller Genetic Risk in Catabolic Process Explains Lower Energy Expenditure, More Athletic Capability and Higher Prevalence of Obesity in Africans
Source: PLoS One. 2011 Oct 10;6(10):e26027. doi: 10.1371/journal.pone.0026027 (PMC3189926; doi:10.1371/journal.pone.0026027)
Supplement: Table S1 — 182 candidate genes in catabolism process with possible harmful variants. (DOC) [file pone.0026027.s017.doc]

Table S1. 182 candidate genes in catabolism process with possible harmful variants

| chr# | symbol | Gene full name | Gene location |
| --- | --- | --- | --- |
| 1 | AGL | amylo-alpha-1, 6-glucosidase, 4-alpha-glucanotransferase | 1p21 |
| 1 | ALDH4A1 | aldehyde dehydrogenase 4 family, member A1 | 1p36 |
| 1 | ARHGEF16 | Rho guanine nucleotide exchange factor (GEF) 16 | 1p36.3 |
| 1 | ASAP3 | ArfGAP with SH3 domain, ankyrin repeat and PH domain 3 | 1p36.12 |
| 1 | CHD1L | chromodomain helicase DNA binding protein 1-like | 1q12 |
| 1 | CHI3L1 | chitinase 3-like 1 (cartilage glycoprotein-39) | 1q32.1 |
| 1 | CHI3L2 | chitinase 3-like 2 | 1p13.3 |
| 1 | CHIA | chitinase, acidic | 1p13.2 |
| 1 | CPT2 | carnitine palmitoyltransferase 2 | 1p32 |
| 1 | CTBS | chitobiase, di-N-acetyl- | 1p22 |
| 1 | DPYD | dihydropyrimidine dehydrogenase | 1p22 |
| 1 | FBXO2 | F-box protein 2 | 1p36.22 |
| 1 | GNAT2 | guanine nucleotide binding protein (G protein), alpha transducing activity polypeptide 2 | 1p13.1 |
| 1 | H6PD | hexose-6-phosphate dehydrogenase (glucose 1-dehydrogenase) | 1p36 |
| 1 | KIF1B | kinesin family member 1B | 1p36.2 |
| 1 | MUTYH | mutY homolog (E. coli) | 1p34.1 |
| 1 | OVGP1 | oviductal glycoprotein 1, 120kDa | 1p13 |
| 1 | PGLYRP3 | peptidoglycan recognition protein 3 | 1q21 |
| 1 | PGLYRP4 | peptidoglycan recognition protein 4 | 1q21 |
| 1 | PSMB4 | proteasome (prosome, macropain) subunit, beta type, 4 | 1q21 |
| 1 | SCP2 | sterol carrier protein 2 | 1p32 |
| 1 | SMG7 | Smg-7 homolog, nonsense mediated mRNA decay factor (C. elegans) | 1q25 |
| 1 | SMPDL3B | sphingomyelin phosphodiesterase, acid-like 3B | 1p35.3 |
| 1 | SYDE2 | synapse defective 1, Rho GTPase, homolog 2 (C. elegans) | 1p22.3 |
| 1 | TNNT2 | troponin T type 2 (cardiac) | 1q32 |
| 1 | USP24 | ubiquitin specific peptidase 24 | 1p32.3 |
| 2 | ABCG8 | ATP-binding cassette, sub-family G (WHITE), member 8 | 2p21 |
| 2 | ACADL | acyl-CoA dehydrogenase, long chain | 2q34-q35 |
| 2 | ACOXL | acyl-CoA oxidase-like | 2q13 |
| 2 | APOB | apolipoprotein B (including Ag(x) antigen) | 2p24-p23 |
| 2 | ATG9A | ATG9 autophagy related 9 homolog A (S. cerevisiae) | 2q35 |
| 2 | FAM176A | family with sequence similarity 176, member A | 2p12 |
| 2 | LPIN1 | lipin 1 | 2p25.1 |
| 2 | MDH1 | malate dehydrogenase 1, NAD (soluble) | 2p13.3 |
| 2 | MDH1B | malate dehydrogenase 1B, NAD (soluble) | 2q33.3 |
| 2 | MSH2 | mutS homolog 2, colon cancer, nonpolyposis type 1 (E. coli) | 2p21 |
| 2 | PLB1 | phospholipase B1 | 2p23.2 |
| 2 | PXDN | peroxidasin homolog (Drosophila) | 2p25 |
| 2 | USP34 | ubiquitin specific peptidase 34 | 2p15 |
| 2 | USP37 | ubiquitin specific peptidase 37 | 2q35 |
| 2 | XDH | xanthine dehydrogenase | 2p23.1 |
| 3 | ADAMTS9 | ADAM metallopeptidase with thrombospondin type 1 motif, 9 | 3p14.1 |
| 3 | CASR | calcium-sensing receptor | 3q13 |
| 3 | CNOT10 | CCR4-NOT transcription complex, subunit 10 | 3p22.3 |
| 3 | MLH1 | mutL homolog 1, colon cancer, nonpolyposis type 2 (E. coli) | 3p21.3 |
| 3 | NR1I2 | nuclear receptor subfamily 1, group I, member 2 | 3q12-q13.3 |
| 3 | OGG1 | 8-oxoguanine DNA glycosylase | 3p26.2 |
| 3 | PLCD1 | phospholipase C, delta 1 | 3p22-p21.3 |
| 3 | UBA7 | ubiquitin-like modifier activating enzyme 7 | 3p21 |
| 3 | USP4 | ubiquitin specific peptidase 4 (proto-oncogene) | 3p21.3 |
| 4 | HERC5 | hect domain and RLD 5 | 4q22.1 |
| 4 | MANBA | mannosidase, beta A, lysosomal | 4q22-q25 |
| 4 | TBC1D1 | TBC1 (tre-2/USP6, BUB2, cdc16) domain family, member 1 | 4p14 |
| 4 | TBC1D9 | TBC1 domain family, member 9 (with GRAM domain) | 4q31.21 |
| 5 | ALDH7A1 | aldehyde dehydrogenase 7 family, member A1 | 5q31 |
| 5 | AMACR | alpha-methylacyl-CoA racemase | 5p13 |
| 5 | ARSB | arylsulfatase B | 5q11-q13 |
| 5 | DAB2 | disabled homolog 2, mitogen-responsive phosphoprotein (Drosophila) | 5p13 |
| 5 | GFM2 | G elongation factor, mitochondrial 2 | 5q13 |
| 5 | HK3 | hexokinase 3 (white cell) | 5q35.2 |
| 5 | HSD17B4 | hydroxysteroid (17-beta) dehydrogenase 4 | 5q21 |
| 5 | LYSMD3 | LysM, putative peptidoglycan-binding, domain containing 3 | 5q14.3 |
| 5 | MAP3K1 | mitogen-activated protein kinase kinase kinase 1 | 5q11.2 |
| 5 | SQSTM1 | sequestosome 1 | 5q35 |
| 6 | ALDH5A1 | aldehyde dehydrogenase 5 family, member A1 | 6p22 |
| 6 | CLPS | colipase, pancreatic | 6pter-p21.1 |
| 6 | CYP39A1 | cytochrome P450, family 39, subfamily A, polypeptide 1 | 6p21.1-p11.2 |
| 6 | ENPP3 | ectonucleotide pyrophosphatase/phosphodiesterase 3 | 6q22 |
| 6 | FBXO5 | F-box protein 5 | 6q25.2 |
| 6 | MDN1 | MDN1, midasin homolog (yeast) | 6q15 |
| 6 | PLA2G7 | phospholipase A2, group VII (platelet-activating factor acetylhydrolase, plasma) | 6p21.2-p12 |
| 6 | SMAP1 | small ArfGAP 1 | 6q13 |
| 6 | TBC1D7 | TBC1 domain family, member 7 | 6p24.1 |
| 6 | TRERF1 | transcriptional regulating factor 1 | 6p21.1-p12.1 |
| 6 | UBD | ubiquitin D | 6p21.3 |
| 6 | USP45 | ubiquitin specific peptidase 45 | 6q16.2 |
| 7 | ACHE | acetylcholinesterase | 7q22 |
| 7 | DAGLB | diacylglycerol lipase, beta | 7p22.1 |
| 7 | HYAL4 | hyaluronoglucosaminidase 4 | 7q31.3 |
| 7 | MGAM | maltase-glucoamylase (alpha-glucosidase) | 7q34 |
| 7 | NOS3 | nitric oxide synthase 3 (endothelial cell) | 7q36 |
| 7 | NUDT1 | nudix (nucleoside diphosphate linked moiety X)-type motif 1 | 7p22 |
| 7 | PION | pigeon homolog (Drosophila) | 7q11.23 |
| 7 | PON1 | paraoxonase 1 | 7q21.3 |
| 7 | PTPRN2 | protein tyrosine phosphatase, receptor type, N polypeptide 2 | 7q36 |
| 8 | ENPP2 | ectonucleotide pyrophosphatase/phosphodiesterase 2 | 8q24.1 |
| 8 | EPHX2 | epoxide hydrolase 2, cytoplasmic | 8p21 |
| 8 | FUT10 | fucosyltransferase 10 (alpha (1,3) fucosyltransferase) | 8p12 |
| 8 | POP1 | processing of precursor 1, ribonuclease P/MRP subunit (S. cerevisiae) | 8q22.1 |
| 8 | SCRIB | scribbled homolog (Drosophila) | 8q24.3 |
| 8 | WDR67 | WD repeat domain 67 | 8q24.13 |
| 8 | WRN | Werner syndrome, RecQ helicase-like | 8p12 |
| 8 | ZHX2 | zinc fingers and homeoboxes 2 | 8q24.13 |
| 9 | DBH | dopamine beta-hydroxylase (dopamine beta-monooxygenase) | 9q34 |
| 9 | EXOSC3 | exosome component 3 | 9p11 |
| 9 | PPP2R4 | protein phosphatase 2A activator, regulatory subunit 4 | 9q34 |
| 9 | SARDH | sarcosine dehydrogenase | 9q33-q34 |
| 10 | CYP2C8 | cytochrome P450, family 2, subfamily C, polypeptide 8 | 10q23.33 |
| 10 | CYP2C9 | cytochrome P450, family 2, subfamily C, polypeptide 9 | 10q24 |
| 10 | ECD | ecdysoneless homolog (Drosophila) | 10q22.3 |
| 10 | KIF20B | kinesin family member 20B | 10q23.31 |
| 10 | LIPF | lipase, gastric | 10q23.31 |
| 10 | PLCE1 | phospholipase C, epsilon 1 | 10q23 |
| 11 | AMPD3 | adenosine monophosphate deaminase 3 | 11p15 |
| 11 | APOA5 | apolipoprotein A-V | 11q23 |
| 11 | ARRB1 | arrestin, beta 1 | 11q13 |
| 11 | ATM | ataxia telangiectasia mutated | 11q22-q23 |
| 11 | DLAT | dihydrolipoamide S-acetyltransferase | 11q23.1 |
| 11 | LYVE1 | lymphatic vessel endothelial hyaluronan receptor 1 | 11p15 |
| 11 | PSMD13 | proteasome (prosome, macropain) 26S subunit, non-ATPase, 13 | 11p15.5 |
| 11 | SMPD1 | sphingomyelin phosphodiesterase 1, acid lysosomal | 11p15.4-p15.1 |
| 12 | CDKN1B | cyclin-dependent kinase inhibitor 1B (p27, Kip1) | 12p13.1-p12 |
| 12 | FGD6 | FYVE, RhoGEF and PH domain containing 6 | 12q22 |
| 12 | GNB3 | guanine nucleotide binding protein (G protein), beta polypeptide 3 | 12p13 |
| 12 | LYZ | lysozyme | 12q15 |
| 12 | NOS1 | nitric oxide synthase 1 (neuronal) | 12q24.2-q24.31 |
| 12 | P2RX7 | purinergic receptor P2X, ligand-gated ion channel, 7 | 12q24 |
| 12 | PLBD1 | phospholipase B domain containing 1 | 12p13.1 |
| 12 | PLBD2 | phospholipase B domain containing 2 | 12q24.13 |
| 12 | PSMD9 | proteasome (prosome, macropain) 26S subunit, non-ATPase, 9 | 12q24.31-q24.32 |
| 12 | SCARB1 | scavenger receptor class B, member 1 | 12q24.31 |
| 13 | FAM48A | family with sequence similarity 48, member A | 13q13.3 |
| 13 | TBC1D4 | TBC1 domain family, member 4 | 13q22.2 |
| 13 | USPL1 | ubiquitin specific peptidase like 1 | 13q12-q14 |
| 14 | ACIN1 | apoptotic chromatin condensation inducer 1 | 14q11.2 |
| 14 | HECTD1 | HECT domain containing 1 | 14q12 |
| 14 | OXA1L | oxidase (cytochrome c) assembly 1-like | 14q11.2 |
| 14 | PSMB5 | proteasome (prosome, macropain) subunit, beta type, 5 | 14q11.2 |
| 14 | PYGL | phosphorylase, glycogen, liver | 14q21-q22 |
| 14 | SLC25A21 | solute carrier family 25 (mitochondrial oxodicarboxylate carrier), member 21 | 14q11.2 |
| 15 | BLM | Bloom syndrome, RecQ helicase-like | 15q26.1 |
| 15 | DUOX2 | dual oxidase 2 | 15q15.3 |
| 15 | DYX1C1 | dyslexia susceptibility 1 candidate 1 | 15q21.3 |
| 15 | LYSMD2 | LysM, putative peptidoglycan-binding, domain containing 2 | 15q21.2 |
| 15 | LYSMD4 | LysM, putative peptidoglycan-binding, domain containing 4 | 15q26.3 |
| 15 | NEDD4 | neural precursor cell expressed, developmentally down-regulated 4 | 15q |
| 15 | PLA2G4E | phospholipase A2, group IVE | 15q15.1 |
| 15 | PLIN1 | perilipin 1 | 15q26 |
| 15 | TBC1D21 | TBC1 domain family, member 21 | 15q24.1 |
| 15 | USP8 | ubiquitin specific peptidase 8 | 15q21.2 |
| 16 | ABCC1 | ATP-binding cassette, sub-family C (CFTR/MRP), member 1 | 16p13.1 |
| 16 | DNASE1 | deoxyribonuclease I | 16p13.3 |
| 16 | ERCC4 | excision repair cross-complementing rodent repair deficiency, complementation group 4 | 16p13.12 |
| 16 | PLCG2 | phospholipase C, gamma 2 (phosphatidylinositol-specific) | 16q24.1 |
| 16 | SLX4 | SLX4 structure-specific endonuclease subunit homolog (S. cerevisiae) | 16p13.3 |
| 16 | SPG7 | spastic paraplegia 7 (pure and complicated autosomal recessive) | 16q24.3 |
| 16 | USP31 | ubiquitin specific peptidase 31 | 16p12.2 |
| 17 | 4-Sep | septin 4 | 17q23 |
| 17 | ACADVL | acyl-CoA dehydrogenase, very long chain | 17p13.1 |
| 17 | ACE | angiotensin I converting enzyme (peptidyl-dipeptidase A) 1 | 17q23.3 |
| 17 | ACLY | ATP citrate lyase | 17q21.2 |
| 17 | GAA | glucosidase, alpha; acid | 17q25.2-q25.3 |
| 17 | GRIN2C | glutamate receptor, ionotropic, N-methyl D-aspartate 2C | 17q25 |
| 17 | MAPT | microtubule-associated protein tau | 17q21.1 |
| 17 | NF1 | neurofibromin 1 | 17q11.2 |
| 17 | NOS2 | nitric oxide synthase 2, inducible | 17q11.2-q12 |
| 17 | NSF | N-ethylmaleimide-sensitive factor | 17q21 |
| 17 | SCARF1 | scavenger receptor class F, member 1 | 17p13.3 |
| 17 | SMG6 | Smg-6 homolog, nonsense mediated mRNA decay factor (C. elegans) | 17p13.3 |
| 17 | TBC1D26 | TBC1 domain family, member 26 | 17p11.2 |
| 17 | USP36 | ubiquitin specific peptidase 36 | 17q25.3 |
| 17 | USP6 | ubiquitin specific peptidase 6 (Tre-2 oncogene) | 17p13 |
| 18 | ATP8B1 | ATPase, aminophospholipid transporter, class I, type 8B, member 1 | 18q21-q22|18q21.31 |
| 19 | APOE | apolipoprotein E | 19q13.2 |
| 19 | ATP8B3 | ATPase, aminophospholipid transporter, class I, type 8B, member 3 | 19p13.3 |
| 19 | CYP2B6 | cytochrome P450, family 2, subfamily B, polypeptide 6 | 19q13.2 |
| 19 | DHDH | dihydrodiol dehydrogenase (dimeric) | 19q13.3 |
| 19 | FUT2 | fucosyltransferase 2 (secretor status included) | 19q13.3 |
| 19 | INSR | insulin receptor | 19p13.3-p13.2 |
| 19 | LONP1 | lon peptidase 1, mitochondrial | 19p13.2 |
| 19 | PLA2G4C | phospholipase A2, group IVC (cytosolic, calcium-independent) | 19q13.3 |
| 19 | PRODH2 | proline dehydrogenase (oxidase) 2 | 19q13.1 |
| 19 | TGFB1 | transforming growth factor, beta 1 | 19q13.2|19q13.1 |
| 19 | USP29 | ubiquitin specific peptidase 29 | 19q13.43 |
| 20 | RALGAPA2 | Ral GTPase activating protein, alpha subunit 2 (catalytic) | 20p11.22 |
| 20 | TGM3 | transglutaminase 3 (E polypeptide, protein-glutamine-gamma-glutamyltransferase) | 20q11.2 |
| 22 | APOBEC3F | apolipoprotein B mRNA editing enzyme, catalytic polypeptide-like 3F | 22q13.1 |
| 22 | APOBEC3G | apolipoprotein B mRNA editing enzyme, catalytic polypeptide-like 3G | 22q13.1-q13.2 |
| 22 | ARSA | arylsulfatase A | 22q13.31-qter|22q13.33 |
| 22 | CECR1 | cat eye syndrome chromosome region, candidate 1 | 22q11.2 |
| 22 | CPT1B | carnitine palmitoyltransferase 1B (muscle) | 22q13.33 |
| X | G6PD | glucose-6-phosphate dehydrogenase | Xq28 |
