# Supplementary material for: Smaller Genetic Risk in Catabolic Process Explains Lower Energy Expenditure, More Athletic Capability and Higher Prevalence of Obesity in Africans
Source: PLoS One. 2011 Oct 10;6(10):e26027. doi: 10.1371/journal.pone.0026027 (PMC3189926; doi:10.1371/journal.pone.0026027)
Supplement: Table S2 — 231 common (MAF>0.05) SNPs screened with possible harmful missense mutations (ri>0.2) on genes in catabolism process. (DOC) [file pone.0026027.s018.doc]

Table S2. 231 common (MAF>0.05) SNPs screened with possible harmful missense mutations (r_i_>0.2) on genes in catabolism process ^*^

| **Chromo-some#** | **SNP#** | **Groups with this SNP** | **Gene symbol** | **r_i_** |
| --- | --- | --- | --- | --- |
| 1 | rs3753494 | ASW,CEU,GIH,LWK,MEX,MKK,TSI,YRI | AGL | 0.967 |
| 1 | rs28730701 | MEX,MKK | AGL | 0.27 |
| 1 | rs6695033 | ASW,LWK,MKK,YRI | ALDH4A1 | 0.767 |
| 1 | rs2185639 | ASW,CEU,CHB,CHD,GIH,JPT,LWK,MEX,MKK,TSI,YRI | ARHGEF16 | 0.744 |
| 1 | rs16828486 | ASW,LWK,YRI | ASAP3 | 0.938 |
| 1 | rs2275249 | CEU,CHB,CHD,GIH,JPT,MEX,MKK,TSI | CHD1L | 0.987 |
| 1 | rs880633 | ASW,CEU,CHB,CHD,GIH,JPT,LWK,MEX,MKK,TSI,YRI | CHI3L1 | 0.975 |
| 1 | rs11556868 | CEU,GIH,MKK,TSI | CHI3L2 | 1 |
| 1 | rs2275254 | ASW,CEU,CHB,CHD,GIH,JPT,LWK,MEX,MKK,TSI,YRI | CHIA | 1 |
| 1 | rs3818822 | ASW,CEU,CHB,CHD,GIH,JPT,LWK,MEX,MKK,TSI,YRI | CHIA | 0.942 |
| 1 | rs1799822 | ASW,CEU,CHB,CHD,GIH,JPT,MEX,MKK,TSI | CPT2 | 0.769 |
| 1 | rs2229291 | CHB,CHD,JPT,MEX | CPT2 | 0.999 |
| 1 | rs15911 | ASW,GIH,LWK,MKK,TSI,YRI | CTBS | 0.953 |
| 1 | rs17376848 | ASW,CEU,CHB,CHD,JPT,MEX | DPYD | 0.998 |
| 1 | rs2297595 | ASW,CEU,LWK,MEX,MKK,TSI | DPYD | 0.999 |
| 1 | rs9614 | ASW,CEU,CHB,CHD,GIH,JPT,LWK,MEX,MKK,TSI,YRI | FBXO2 | 0.985 |
| 1 | rs3738766 | CHB,CHD,GIH,JPT,MEX,TSI | GNAT2 | 0.704 |
| 1 | rs17368528 | CEU,CHB,CHD,GIH,JPT,MEX,MKK,TSI | H6PD | 0.966 |
| 1 | rs2297881 | CHB,JPT,LWK | KIF1B | 0.994 |
| 1 | rs3219484 | CEU | MUTYH | 0.728 |
| 1 | rs3767607 | ASW,CEU,CHB,CHD,GIH,JPT,LWK,MEX,MKK,TSI,YRI | OVGP1 | 0.931 |
| 1 | rs954739 | ASW,CEU,CHB,CHD,GIH,JPT,MEX,MKK,TSI,YRI | OVGP1 | 0.531 |
| 1 | rs17027633 | CHB,CHD,GIH,JPT | OVGP1 | 0.999 |
| 1 | rs843971 | ASW,CEU,CHB,CHD,GIH,JPT,LWK,MEX,MKK,TSI,YRI | PGLYRP3 | 0.997 |
| 1 | rs3014863 | CEU,CHB,CHD,GIH,JPT,MEX,TSI | PGLYRP4 | 0.992 |
| 1 | rs3006453 | GIH,MEX,TSI | PGLYRP4 | 0.719 |
| 1 | rs4603 | ASW,CEU,CHB,CHD,GIH,JPT,LWK,MEX,MKK,TSI,YRI | PSMB4 | 0.336 |
| 1 | rs17107531 | YRI | SCP2 | 1 |
| 1 | rs2298083 | ASW,CEU,CHB,CHD,GIH,JPT,LWK,MEX,MKK,TSI,YRI | SMG7 | 0.25 |
| 1 | rs3813803 | ASW,CEU,CHB,CHD,GIH,JPT,LWK,MEX,MKK,TSI,YRI | SMPDL3B | 0.656 |
| 1 | rs17122989 | ASW,LWK,MKK,YRI | SYDE2 | 0.463 |
| 1 | rs4523540 | MKK | TNNT2 | 0.838 |
| 1 | rs17410294 | ASW,CEU,GIH,TSI | USP24 | 0.991 |
| 2 | rs6756629 | ASW,CEU,LWK,MEX,MKK,TSI,YRI | ABCG8 | 0.985 |
| 2 | rs2286963 | ASW,CEU,CHB,CHD,GIH,JPT,LWK,MEX,MKK,TSI,YRI | ACADL | 0.942 |
| 2 | rs17041850 | CHB,CHD,JPT,MEX | ACOXL | 0.47 |
| 2 | rs1042031 | ASW,CEU,GIH,LWK,MEX,MKK,TSI,YRI | APOB | 0.445 |
| 2 | rs12720847 | ASW,LWK,MKK,YRI | APOB | 0.686 |
| 2 | rs12720855 | ASW,LWK,YRI | APOB | 0.914 |
| 2 | rs676210 | ASW,CEU,CHB,CHD,GIH,JPT,LWK,MEX,MKK,TSI,YRI | APOB | 0.894 |
| 2 | rs1801700 | CEU | APOB | 0.686 |
| 2 | rs13306198 | CHB,CHD | APOB | 0.858 |
| 2 | rs533617 | TSI | APOB | 0.975 |
| 2 | rs2276635 | CEU,GIH,MEX,MKK,TSI | ATG9A | 0.33 |
| 2 | rs11126472 | ASW,CEU,TSI,YRI | FAM176A | 0.999 |
| 2 | rs4669781 | GIH,MEX | LPIN1 | 0.309 |
| 2 | rs11555102 | TSI | MDH1 | 0.986 |
| 2 | rs2287632 | ASW,CEU,GIH,JPT,LWK,MEX,MKK,TSI,YRI | MDH1B | 0.27 |
| 2 | rs17217772 | ASW,LWK,MKK,YRI | MSH2 | 0.356 |
| 2 | rs2199619 | ASW,CEU,CHB,CHD,GIH,JPT,LWK,MEX,MKK,TSI,YRI | PLB1 | 0.993 |
| 2 | rs1863135 | ASW,CEU,LWK,MKK,TSI,YRI | PXDN | 0.637 |
| 2 | rs6723697 | ASW,LWK | PXDN | 1 |
| 2 | rs6723818 | ASW,LWK,YRI | USP34 | 0.831 |
| 2 | rs6436058 | ASW,LWK,YRI | USP37 | 0.981 |
| 2 | rs17011368 | ASW,LWK,MKK,TSI,YRI | XDH | 0.336 |
| 3 | rs6771712 | ASW,CEU,LWK,MEX,MKK,TSI,YRI | ADAMTS9 | 0.307 |
| 3 | rs1042636 | CEU,CHB,CHD,GIH,JPT,MEX,TSI | CASR | 0.939 |
| 3 | rs11558687 | YRI | CNOT10 | 0.478 |
| 3 | rs2020873 | ASW,LWK,YRI | MLH1 | 0.988 |
| 3 | rs12721608 | MKK | NR1I2 | 1 |
| 3 | rs1805373 | ASW,LWK,YRI | OGG1 | 0.252 |
| 3 | rs933135 | CHB,CHD,GIH,JPT | PLCD1 | 0.822 |
| 3 | rs2230149 | ASW,LWK,MKK,YRI | UBA7 | 1 |
| 3 | rs9311440 | LWK,YRI | USP4 | 1 |
| 4 | rs17014143 | ASW,LWK,YRI | HERC5 | 0.899 |
| 4 | rs2272697 | ASW,CEU,CHB,CHD,GIH,JPT,LWK,MEX,MKK,TSI,YRI | MANBA | 0.688 |
| 4 | rs13110318 | CEU,CHB,CHD,JPT,LWK,MEX,TSI,YRI | TBC1D1 | 0.282 |
| 4 | rs1560440 | ASW,CEU,CHB,CHD,GIH,JPT,LWK,MEX,MKK,TSI,YRI | TBC1D9 | 0.897 |
| 5 | rs2306618 | CHB,CHD,JPT | ALDH7A1 | 0.863 |
| 5 | rs10941112 | ASW,CEU,CHB,CHD,GIH,JPT,MEX,TSI | AMACR | 0.244 |
| 5 | rs34677 | CEU,CHB,CHD,GIH,JPT,MEX,MKK,TSI,YRI | AMACR | 0.342 |
| 5 | rs1065757 | ASW,CEU,CHB,CHD,GIH,JPT,MEX,MKK,TSI | ARSB | 0.987 |
| 5 | rs3733801 | ASW,CEU,CHD,GIH,JPT,MEX,MKK,TSI | DAB2 | 0.656 |
| 5 | rs16872235 | ASW,CEU,GIH,LWK,MEX,MKK,TSI,YRI | GFM2 | 0.463 |
| 5 | rs2278492 | ASW,CEU,CHB,CHD,GIH,JPT,LWK,MEX,MKK,TSI,YRI | HK3 | 0.455 |
| 5 | rs25640 | ASW,CEU,CHB,CHD,GIH,JPT,LWK,MEX,MKK,TSI,YRI | HSD17B4 | 0.439 |
| 5 | rs10069050 | ASW,CEU,CHB,CHD,GIH,JPT,LWK,MEX,MKK,TSI,YRI | LYSMD3 | 0.968 |
| 5 | rs702689 | ASW,CEU,CHB,CHD,GIH,JPT,LWK,MEX,MKK,TSI,YRI | MAP3K1 | 0.968 |
| 5 | rs10277 | ASW,CEU,CHB,CHD,GIH,JPT,LWK,MEX,MKK,TSI,YRI | SQSTM1 | 0.992 |
| 6 | rs3765310 | CHB,CHD,GIH,JPT,LWK,MKK | ALDH5A1 | 0.823 |
| 6 | rs2766597 | ASW,LWK,MKK,YRI | CLPS | 0.339 |
| 6 | rs12192544 | ASW,CEU,CHB,CHD,GIH,JPT,MEX,MKK,TSI | CYP39A1 | 0.936 |
| 6 | rs17601580 | CEU,GIH,MEX,MKK,TSI | ENPP3 | 0.576 |
| 6 | rs7763565 | ASW,GIH,LWK,MEX,MKK,TSI,YRI | FBXO5 | 0.486 |
| 6 | rs12530146 | ASW,CHB,CHD,GIH,JPT,LWK,MKK,TSI,YRI | MDN1 | 0.98 |
| 6 | rs9294445 | ASW,CEU,CHB,CHD,GIH,JPT,LWK,MEX,MKK,TSI,YRI | MDN1 | 0.986 |
| 6 | rs1051931 | MKK,TSI,YRI | PLA2G7 | 0.92 |
| 6 | rs2273566 | ASW,CEU,CHB,CHD,GIH,JPT,LWK,MEX,MKK,TSI,YRI | SMAP1 | 0.205 |
| 6 | rs543580 | ASW,LWK,YRI | TBC1D7 | 0.813 |
| 6 | rs7744694 | ASW,LWK,MKK,YRI | TRERF1 | 0.307 |
| 6 | rs11751765 | TSI | TRERF1 | 0.968 |
| 6 | rs2076484 | ASW,CHB,CHD,GIH,JPT,LWK,MEX,MKK,TSI,YRI | UBD | 0.418 |
| 6 | rs2076485 | CEU,CHB,CHD,GIH,JPT,MEX,MKK,TSI | UBD | 0.999 |
| 6 | rs7744845 | ASW,CEU,CHB,CHD,GIH,JPT,LWK,MEX,MKK,TSI | USP45 | 0.993 |
| 7 | rs1799805 | CEU,TSI | ACHE | 0.397 |
| 7 | rs8286 | MKK | ACHE | 1 |
| 7 | rs2303361 | ASW,CEU,CHB,CHD,GIH,JPT,MEX,TSI,YRI | DAGLB | 0.293 |
| 7 | rs12672205 | CHB,CHD,JPT,MEX | HYAL4 | 0.982 |
| 7 | rs10266732 | ASW,LWK,MKK,YRI | MGAM | 0.465 |
| 7 | rs9655651 | LWK | MGAM | 0.977 |
| 7 | rs3918166 | ASW,LWK,MKK,YRI | NOS3 | 0.615 |
| 7 | rs4866 | JPT | NUDT1 | 0.992 |
| 7 | rs17151689 | ASW,CHB,CHD,GIH,LWK,MKK,YRI | PION | 0.999 |
| 7 | rs13306698 | CHB,CHD,JPT | PON1 | 0.709 |
| 7 | rs1130499 | ASW,CEU,CHB,CHD,GIH,JPT,LWK,MEX,MKK,TSI,YRI | PTPRN2 | 0.438 |
| 7 | rs3752368 | CHB,CHD,JPT | PTPRN2 | 0.205 |
| 7 | rs1130496 | CHD,GIH,MKK | PTPRN2 | 0.802 |
| 8 | rs1058913 | ASW,CEU,CHB,GIH,MEX,TSI | ENPP2 | 0.852 |
| 8 | rs2305129 | ASW,CHB,CHD,GIH,JPT,LWK,MEX,MKK,TSI,YRI | ENPP2 | 1 |
| 8 | rs4871364 | ASW,CEU,CHB,CHD,GIH,JPT,LWK,MEX,MKK,TSI,YRI | ENPP2 | 0.999 |
| 8 | rs17057255 | ASW,LWK,MKK,YRI | EPHX2 | 0.853 |
| 8 | rs751141 | ASW,CEU,CHB,CHD,GIH,JPT,LWK,MEX,MKK,TSI,YRI | EPHX2 | 1 |
| 8 | rs16880994 | ASW,LWK,YRI | FUT10 | 0.401 |
| 8 | rs17184326 | ASW,CEU,GIH,LWK,MEX,MKK,TSI,YRI | POP1 | 0.836 |
| 8 | rs6558394 | ASW,CEU,CHB,CHD,GIH,JPT,LWK,MEX,MKK,TSI,YRI | SCRIB | 0.998 |
| 8 | rs16898023 | MEX | WDR67 | 0.266 |
| 8 | rs1346044 | ASW,CEU,CHB,CHD,GIH,JPT,LWK,MEX,MKK,TSI,YRI | WRN | 0.999 |
| 8 | rs1800391 | CEU,GIH,MEX,TSI | WRN | 0.79 |
| 8 | rs3802264 | CHB,CHD,GIH,JPT | ZHX2 | 0.927 |
| 9 | rs6271 | CEU,MEX | DBH | 0.974 |
| 9 | rs3208406 | CEU,MEX,TSI | EXOSC3 | 0.956 |
| 9 | rs2480452 | CEU,CHB,CHD,GIH,JPT,MEX,TSI | PPP2R4 | 0.872 |
| 9 | rs573904 | ASW,CEU,CHB,CHD,GIH,JPT,LWK,MEX,MKK,TSI,YRI | SARDH | 0.419 |
| 10 | rs1058930 | CEU | CYP2C8 | 0.951 |
| 10 | rs28371685 | MKK | CYP2C9 | 1 |
| 10 | rs2271904 | ASW,CEU,CHB,CHD,GIH,JPT,LWK,MKK,YRI | ECD | 0.557 |
| 10 | rs1886996 | ASW,CEU,JPT,LWK,MEX,MKK,TSI,YRI | KIF20B | 0.969 |
| 10 | rs814628 | CEU,CHB,CHD,GIH,JPT,MEX,MKK,TSI | LIPF | 0.421 |
| 10 | rs17508082 | TSI | PLCE1 | 0.697 |
| 11 | rs11042836 | CEU,MEX,TSI | AMPD3 | 0.25 |
| 11 | rs2075291 | CHD | APOA5 | 0.61 |
| 11 | rs877711 | ASW,CEU,CHB,CHD,GIH,JPT,LWK,MEX,MKK,TSI,YRI | ARRB1 | 0.285 |
| 11 | rs1801516 | CEU,GIH,MEX,TSI | ATM | 0.251 |
| 11 | rs2235000 | YRI | ATM | 0.365 |
| 11 | rs10891314 | ASW,CEU,CHB,CHD,GIH,JPT,LWK,MEX,MKK,TSI,YRI | DLAT | 0.992 |
| 11 | rs11553595 | ASW,LWK,MKK,YRI | DLAT | 0.658 |
| 11 | rs7948666 | ASW,YRI | LYVE1 | 0.819 |
| 11 | rs28365927 | ASW,CEU,CHB,CHD,GIH,JPT,LWK,MEX,MKK,TSI,YRI | PSMD13 | 0.854 |
| 11 | rs1050239 | ASW,CEU,CHB,CHD,GIH,JPT,LWK,MEX,MKK,TSI,YRI | SMPD1 | 0.735 |
| 12 | rs2066828 | MKK | CDKN1B | 1 |
| 12 | rs10507047 | ASW,CEU,CHB,CHD,GIH,JPT,LWK,MEX,MKK,TSI,YRI | FGD6 | 0.97 |
| 12 | rs5442 | CEU | GNB3 | 0.228 |
| 12 | rs1800973 | TSI | LYZ | 0.962 |
| 12 | rs1047735 | ASW,CEU,CHB,CHD,GIH,JPT,LWK,MEX,MKK,TSI,YRI | NOS1 | 0.972 |
| 12 | rs9658403 | YRI | NOS1 | 0.941 |
| 12 | rs3751143 | ASW,CEU,CHB,CHD,GIH,JPT,LWK,MEX,MKK,TSI,YRI | P2RX7 | 0.985 |
| 12 | rs7958311 | ASW,CEU,CHB,CHD,GIH,JPT,MEX,MKK,TSI | P2RX7 | 0.9 |
| 12 | rs208294 | CEU,CHB,CHD,GIH,JPT,LWK,MEX,MKK,TSI | P2RX7 | 1 |
| 12 | rs2287541 | ASW,CEU,GIH,MEX,TSI | PLBD1 | 0.366 |
| 12 | rs12425042 | ASW,CEU,GIH,MEX,TSI,YRI | PLBD2 | 0.746 |
| 12 | rs1177573 | ASW,LWK,MKK,YRI | PSMD9 | 0.25 |
| 12 | rs5892 | GIH,LWK,MKK,YRI | SCARB1 | 0.996 |
| 13 | rs9469 | ASW,CEU,CHB,CHD,GIH,JPT,LWK,MEX,MKK,TSI,YRI | FAM48A | 0.732 |
| 13 | rs17254379 | CEU,MEX,TSI | TBC1D4 | 0.998 |
| 13 | rs3742303 | CHB,GIH,JPT | USPL1 | 0.999 |
| 14 | rs11555803 | ASW,LWK,MKK,YRI | ACIN1 | 0.956 |
| 14 | rs1885097 | ASW,CEU,CHB,CHD,GIH,JPT,LWK,MEX,MKK,TSI,YRI | ACIN1 | 0.914 |
| 14 | rs3751501 | CEU,CHB,CHD,JPT,TSI | ACIN1 | 0.981 |
| 14 | rs11620816 | ASW,CEU,CHB,CHD,GIH,JPT,LWK,MEX,MKK,TSI,YRI | HECTD1 | 0.514 |
| 14 | rs17619 | ASW,GIH,JPT,LWK,MKK,TSI,YRI | OXA1L | 0.813 |
| 14 | rs11543947 | ASW,CEU,GIH,TSI | PSMB5 | 0.982 |
| 14 | rs946616 | ASW,CEU,GIH,JPT,LWK,MEX,MKK,TSI,YRI | PYGL | 0.336 |
| 14 | rs17104991 | MKK | SLC25A21 | 0.999 |
| 15 | rs11852361 | ASW,LWK,MEX,MKK,YRI | BLM | 0.387 |
| 15 | rs269868 | ASW,CEU,CHB,CHD,JPT,LWK,MEX,MKK,TSI,YRI | DUOX2 | 0.24 |
| 15 | rs600753 | ASW,CEU,CHB,CHD,GIH,JPT,LWK,MEX,MKK,TSI,YRI | DYX1C1 | 0.249 |
| 15 | rs7168775 | ASW,LWK | LYSMD2 | 0.205 |
| 15 | rs3751593 | CHB,CHD,JPT | LYSMD2 | 0.984 |
| 15 | rs2061007 | ASW,CEU,CHB,CHD,GIH,JPT,MEX,TSI,YRI | LYSMD4 | 0.831 |
| 15 | rs2303580 | ASW,CEU,CHB,CHD,GIH,JPT,LWK,MEX,MKK,TSI,YRI | NEDD4 | 0.9 |
| 15 | rs1912403 | CEU,TSI | NEDD4 | 0.978 |
| 15 | rs4924595 | CEU,CHB,CHD,GIH,JPT,TSI | PLA2G4E | 0.862 |
| 15 | rs8179071 | GIH | PLIN1 | 0.998 |
| 15 | rs16958445 | CHB,CHD,GIH,JPT | TBC1D21 | 0.762 |
| 15 | rs3743044 | CHB,GIH,JPT | USP8 | 0.916 |
| 16 | rs13337489 | LWK,YRI | ABCC1 | 0.41 |
| 16 | rs8176919 | ASW,MKK,YRI | DNASE1 | 0.986 |
| 16 | rs8176927 | ASW,LWK,MKK,YRI | DNASE1 | 0.973 |
| 16 | rs2791 | CEU,MEX | DNASE1 | 0.483 |
| 16 | rs1800067 | TSI | ERCC4 | 1 |
| 16 | rs17537869 | CEU,TSI | PLCG2 | 0.998 |
| 16 | rs3810813 | CHB,CHD,JPT,MEX,TSI | SLX4 | 0.679 |
| 16 | rs12960 | ASW,CEU,GIH,MEX | SPG7 | 0.805 |
| 16 | rs10083789 | ASW,CEU,CHB,CHD,GIH,LWK,MEX,MKK,TSI,YRI | USP31 | 0.999 |
| 16 | rs1978066 | CHB,CHD,JPT | USP31 | 0.605 |
| 16 | rs9932912 | LWK,MKK | USP31 | 0.858 |
| 17 | rs17741424 | CEU,GIH,MEX,TSI | 4-Sep | 0.856 |
| 17 | rs13383 | MKK | ACADVL | 0.848 |
| 17 | rs3730043 | LWK,MEX | ACE | 0.964 |
| 17 | rs8065502 | ASW,CEU,LWK,MEX,MKK,TSI,YRI | ACLY | 0.852 |
| 17 | rs1800315 | ASW,GIH,LWK,MKK,YRI | GAA | 0.753 |
| 17 | rs1800307 | CHB,CHD,JPT | GAA | 0.916 |
| 17 | rs3744215 | ASW,CEU,CHB,CHD,GIH,JPT,LWK,MEX,MKK,TSI,YRI | GRIN2C | 0.62 |
| 17 | rs17651549 | ASW,CEU,GIH,MEX,TSI | MAPT | 0.998 |
| 17 | rs11080149 | CEU,GIH,MEX,TSI | NF1 | 0.469 |
| 17 | rs3730017 | ASW,LWK,MEX,MKK,YRI | NOS2 | 0.533 |
| 17 | rs155733 | ASW,CEU,GIH,TSI | NSF | 0.513 |
| 17 | rs3760460 | ASW,CHD | SCARF1 | 0.427 |
| 17 | rs216195 | ASW,CEU,CHB,CHD,GIH,JPT,LWK,MEX,MKK,TSI,YRI | SMG6 | 0.868 |
| 17 | rs11650318 | ASW,CEU,CHB,CHD,GIH,JPT,LWK,MEX,MKK,TSI | TBC1D26 | 0.96 |
| 17 | rs17855672 | ASW,CEU,CHB,CHD,GIH,JPT,LWK,MEX,MKK,TSI,YRI | TBC1D26 | 0.497 |
| 17 | rs3744797 | CHB,JPT | USP36 | 0.836 |
| 17 | rs9899177 | ASW,CEU,CHB,CHD,GIH,JPT,LWK,MEX,MKK,TSI,YRI | USP6 | 0.975 |
| 18 | rs12968116 | ASW,CEU,GIH,MEX,MKK,TSI | ATP8B1 | 0.999 |
| 18 | rs3745078 | CHD,JPT | ATP8B1 | 0.97 |
| 19 | rs7412 | ASW,CHB,CHD,JPT,LWK,MEX,MKK,YRI | APOE | 1 |
| 19 | rs16994559 | ASW,GIH,LWK,MKK,TSI,YRI | ATP8B3 | 0.209 |
| 19 | rs8100856 | ASW,CEU,CHB,CHD,GIH,JPT,LWK,MEX,MKK,TSI,YRI | ATP8B3 | 0.209 |
| 19 | rs28399499 | ASW,LWK,YRI | CYP2B6 | 1 |
| 19 | rs8192709 | JPT,LWK | CYP2B6 | 0.639 |
| 19 | rs3765148 | ASW,CEU,CHB,JPT,LWK,MEX,MKK,TSI,YRI | DHDH | 0.999 |
| 19 | rs602662 | ASW,CEU,GIH,LWK,MEX,MKK,TSI,YRI | FUT2 | 0.954 |
| 19 | rs1799817 | ASW,CEU,CHB,CHD,GIH,JPT,LWK,MEX,MKK,TSI,YRI | INSR | 1 |
| 19 | rs16994192 | CHD,MKK | INSR | 0.905 |
| 19 | rs11085147 | CEU | LONP1 | 0.201 |
| 19 | rs11564620 | ASW,CEU,GIH,LWK,MEX,MKK,TSI,YRI | PLA2G4C | 0.482 |
| 19 | rs2307282 | ASW,LWK,MKK,YRI | PLA2G4C | 0.998 |
| 19 | rs3761097 | CHB,CHD,JPT,MKK,TSI | PRODH2 | 0.871 |
| 19 | rs1800472 | MEX | TGFB1 | 0.855 |
| 19 | rs1027392 | ASW,CEU,CHD,GIH,JPT,LWK,MEX,MKK,TSI,YRI | USP29 | 0.52 |
| 19 | rs3795003 | ASW,CHB,CHD,GIH,JPT,LWK,MEX,MKK,YRI | USP29 | 0.789 |
| 20 | rs2273528 | ASW,CHB,CHD,JPT,LWK,MEX,MKK,YRI | RALGAPA2 | 1 |
| 20 | rs6048066 | ASW,LWK,MKK,YRI | TGM3 | 0.979 |
| 22 | rs2076101 | ASW,CEU,CHB,CHD,GIH,JPT,LWK,MEX,MKK,TSI,YRI | APOBEC3F | 0.839 |
| 22 | rs8177832 | ASW,CHB,CHD,JPT,LWK,MKK,YRI | APOBEC3G | 0.393 |
| 22 | rs6151415 | CEU,TSI | ARSA | 0.997 |
| 22 | rs362129 | ASW,CEU,GIH,JPT,LWK,MEX,MKK,TSI,YRI | CECR1 | 0.428 |
| 22 | rs2269383 | ASW,CHB,CHD,JPT,LWK,MKK,YRI | CPT1B | 0.999 |
| 22 | rs470117 | ASW,CEU,CHB,CHD,GIH,JPT,LWK,MEX,MKK,TSI,YRI | CPT1B | 0.548 |
| X | rs1050828 | ASW,LWK,YRI | G6PD | 0.418 |
| X | rs2230036 | LWK,MKK | G6PD | 1 |

*Values in the r column are the HumDiv-trained harmful probability of missense mutation at each SNP estimated by Polyphen-2 (http://genetics.bwh.harvard.edu/pph2/dbsearch.shtml).
