# Supplementary material for: Smaller Genetic Risk in Catabolic Process Explains Lower Energy Expenditure, More Athletic Capability and Higher Prevalence of Obesity in Africans
Source: PLoS One. 2011 Oct 10;6(10):e26027. doi: 10.1371/journal.pone.0026027 (PMC3189926; doi:10.1371/journal.pone.0026027)
Supplement: Table S3 — Minor allele frequencies (MAFs) for 231 common (MAF>0.05) SNPs screened with possibly harmful missense mutations (ri>0.2) on genes in catabolism process. (DOC) [file pone.0026027.s019.doc]

Table S3. Minor allele frequencies (MAFs) for 231 common (MAF>0.05) SNPs screened with possibly harmful missense mutations (r_i_>0.2) on genes in catabolism process ^a^

| Chr# | rs# | ASW | CEU | CHB | CHD | GIH | JPT | LWK | MEX | MKK | TSI | YRI |
| --- | --- | --- | --- | --- | --- | --- | --- | --- | --- | --- | --- | --- |
| 1 | rs3753494 | 0.094 | 0.107 | - | - | 0.129 | - | 0.118 | 0.121 | 0.212 | 0.181 | 0.194 |
| 1 | rs28730701 | - | - | - | - | - | - | - | 0.054 | 0.058 | - | - |
| 1 | rs6695033 | 0.094 | - | - | - | - | - | 0.232 | - | 0.282 | - | 0.214 |
| 1 | rs2185639 | 0.472 | 0.143 | 0.325 | 0.319 | 0.094 | 0.212 | 0.373 | 0.336 | 0.375 | 0.113 | 0.364 |
| 1 | rs16828486 | 0.085 | - | - | - | - | - | 0.078 | - | - | - | 0.133 |
| 1 | rs2275249 | - | 0.099 | 0.168 | 0.193 | 0.170 | 0.204 | - | 0.095 | 0.103 | 0.098 | - |
| 1 | rs880633 | 0.264 | 0.482 | 0.376 | 0.445 | 0.480 | 0.332 | 0.109 | 0.328 | 0.173 | 0.471 | 0.157 |
| 1 | rs11556868 | - | 0.103 | - | - | 0.218 | - | - | - | 0.058 | 0.123 | - |
| 1 | rs2275254 | 0.382 | 0.380 | 0.230 | 0.329 | 0.384 | 0.433 | 0.266 | 0.353 | 0.281 | 0.328 | 0.303 |
| 1 | rs3818822 | 0.198 | 0.117 | 0.117 | 0.119 | 0.130 | 0.071 | 0.106 | 0.086 | 0.119 | 0.103 | 0.208 |
| 1 | rs1799822 | 0.085 | 0.183 | 0.066 | 0.069 | 0.094 | 0.067 | - | 0.228 | 0.054 | 0.230 | - |
| 1 | rs2229291 | - | - | 0.221 | 0.243 | - | 0.190 | - | 0.052 | - | - | - |
| 1 | rs15911 | 0.128 | - | - | - | 0.347 | - | 0.075 | - | 0.181 | 0.197 | 0.094 |
| 1 | rs17376848 | 0.057 | 0.058 | 0.117 | 0.124 | - | 0.120 | - | 0.069 | - | - | - |
| 1 | rs2297595 | 0.059 | 0.072 | - | - | - | - | 0.115 | 0.079 | 0.068 | 0.104 | - |
| 1 | rs9614 | 0.198 | 0.152 | 0.485 | 0.482 | 0.233 | 0.443 | 0.109 | 0.216 | 0.122 | 0.196 | 0.163 |
| 1 | rs3738766 | - | - | 0.231 | 0.220 | 0.131 | 0.248 | - | 0.053 | - | 0.074 | - |
| 1 | rs17368528 | - | 0.113 | 0.059 | 0.050 | 0.228 | 0.094 | - | 0.140 | 0.058 | 0.139 | - |
| 1 | rs2297881 | - | - | 0.067 | - | - | 0.071 | 0.059 | - | - | - | - |
| 1 | rs3219484 | - | 0.068 | - | - | - | - | - | - | - | - | - |
| 1 | rs3767607 | 0.113 | 0.058 | 0.261 | 0.211 | 0.144 | 0.248 | 0.119 | 0.121 | 0.138 | 0.059 | 0.153 |
| 1 | rs954739 | 0.113 | 0.268 | 0.099 | 0.110 | 0.178 | 0.137 | - | 0.155 | 0.074 | 0.211 | 0.058 |
| 1 | rs17027633 | - | - | 0.178 | 0.156 | 0.144 | 0.205 | - | - | - | - | - |
| 1 | rs843971 | 0.425 | 0.378 | 0.431 | 0.473 | 0.409 | 0.394 | 0.468 | 0.491 | 0.458 | 0.422 | 0.361 |
| 1 | rs3014863 | - | 0.107 | 0.107 | 0.151 | 0.094 | 0.080 | - | 0.181 | - | 0.059 | - |
| 1 | rs3006453 | - | - | - | - | 0.054 | - | - | 0.052 | - | 0.074 | - |
| 1 | rs4603 | 0.057 | 0.125 | 0.357 | 0.431 | 0.183 | 0.326 | 0.050 | 0.328 | 0.118 | 0.167 | 0.068 |
| 1 | rs17107531 | - | - | - | - | - | - | - | - | - | - | 0.051 |
| 1 | rs2298083 | 0.264 | 0.098 | 0.113 | 0.087 | 0.094 | 0.121 | 0.326 | 0.190 | 0.164 | 0.074 | 0.276 |
| 1 | rs3813803 | 0.104 | 0.250 | 0.287 | 0.250 | 0.267 | 0.239 | 0.114 | 0.207 | 0.087 | 0.245 | 0.051 |
| 1 | rs17122989 | 0.255 | - | - | - | - | - | 0.155 | - | 0.263 | - | 0.164 |
| 1 | rs4523540 | - | - | - | - | - | - | - | - | 0.207 | - | - |
| 1 | rs17410294 | 0.066 | 0.152 | - | - | 0.074 | - | - | - | - | 0.162 | - |
| 2 | rs6756629 | 0.094 | 0.076 | - | - | - | - | 0.123 | 0.138 | 0.115 | 0.069 | 0.071 |
| 2 | rs2286963 | 0.189 | 0.353 | 0.208 | 0.216 | 0.297 | 0.195 | 0.100 | 0.285 | 0.077 | 0.324 | 0.095 |
| 2 | rs17041850 | - | - | 0.235 | 0.252 | - | 0.266 | - | 0.070 | - | - | - |
| 2 | rs1042031 | 0.160 | 0.200 | - | - | 0.094 | - | 0.214 | 0.181 | 0.087 | 0.172 | 0.167 |
| 2 | rs12720847 | 0.094 | - | - | - | - | - | 0.068 | - | 0.103 | - | 0.065 |
| 2 | rs12720855 | 0.057 | - | - | - | - | - | 0.105 | - | - | - | 0.090 |
| 2 | rs676210 | 0.198 | 0.205 | 0.294 | 0.248 | 0.475 | 0.295 | 0.132 | 0.224 | 0.099 | 0.250 | 0.146 |
| 2 | rs1801700 | - | 0.054 | - | - | - | - | - | - | - | - | - |
| 2 | rs13306198 | - | - | 0.066 | 0.055 | - | - | - | - | - | - | - |
| 2 | rs533617 | - | - | - | - | - | - | - | - | - | 0.054 | - |
| 2 | rs2276635 | - | 0.098 | - | - | 0.218 | - | - | 0.129 | 0.074 | 0.093 | - |
| 2 | rs11126472 | 0.085 | 0.063 | - | - | - | - | - | - | - | 0.083 | 0.109 |
| 2 | rs4669781 | - | - | - | - | 0.079 | - | - | 0.069 | - | - | - |
| 2 | rs11555102 | - | - | - | - | - | - | - | - | - | 0.057 | - |
| 2 | rs2287632 | 0.160 | 0.054 | - | - | 0.069 | 0.116 | 0.211 | 0.095 | 0.115 | 0.059 | 0.157 |
| 2 | rs17217772 | 0.057 | - | - | - | - | - | 0.064 | - | 0.138 | - | 0.099 |
| 2 | rs2199619 | 0.250 | 0.277 | 0.329 | 0.358 | 0.253 | 0.237 | 0.243 | 0.204 | 0.148 | 0.284 | 0.185 |
| 2 | rs1863135 | 0.113 | 0.098 | - | - | - | - | 0.073 | - | 0.061 | 0.074 | 0.137 |
| 2 | rs6723697 | 0.057 | - | - | - | - | - | 0.087 | - | - | - | - |
| 2 | rs6723818 | 0.057 | - | - | - | - | - | 0.114 | - | - | - | 0.078 |
| 2 | rs6436058 | 0.057 | - | - | - | - | - | 0.124 | - | - | - | 0.061 |
| 2 | rs17011368 | 0.142 | - | - | - | - | - | 0.159 | - | 0.164 | 0.054 | 0.112 |
| 3 | rs6771712 | 0.066 | 0.063 | - | - | - | - | 0.105 | 0.069 | 0.138 | 0.064 | 0.133 |
| 3 | rs1042636 | - | 0.076 | 0.496 | 0.495 | 0.267 | 0.465 | - | 0.285 | - | 0.059 | - |
| 3 | rs11558687 | - | - | - | - | - | - | - | - | - | - | 0.058 |
| 3 | rs2020873 | 0.087 | - | - | - | - | - | 0.136 | - | - | - | 0.162 |
| 3 | rs12721608 | - | - | - | - | - | - | - | - | 0.190 | - | - |
| 3 | rs1805373 | 0.066 | - | - | - | - | - | 0.073 | - | - | - | 0.099 |
| 3 | rs933135 | - | - | 0.199 | 0.225 | 0.114 | 0.219 | - | - | - | - | - |
| 3 | rs2230149 | 0.075 | - | - | - | - | - | 0.128 | - | 0.101 | - | 0.078 |
| 3 | rs9311440 | - | - | - | - | - | - | 0.050 | - | - | - | 0.054 |
| 4 | rs17014143 | 0.104 | - | - | - | - | - | 0.079 | - | - | - | 0.078 |
| 4 | rs2272697 | 0.340 | 0.460 | 0.464 | 0.463 | 0.446 | 0.482 | 0.396 | 0.285 | 0.388 | 0.490 | 0.337 |
| 4 | rs13110318 | - | 0.080 | 0.110 | 0.115 | - | 0.124 | 0.068 | 0.190 | - | 0.098 | 0.065 |
| 4 | rs1560440 | 0.142 | 0.162 | 0.453 | 0.317 | 0.149 | 0.345 | 0.059 | 0.167 | 0.155 | 0.196 | 0.085 |
| 5 | rs2306618 | - | - | 0.062 | 0.078 | - | 0.063 | - | - | - | - | - |
| 5 | rs10941112 | 0.123 | 0.438 | 0.363 | 0.312 | 0.149 | 0.391 | - | 0.447 | - | 0.451 | - |
| 5 | rs34677 | - | 0.143 | 0.146 | 0.136 | 0.188 | 0.111 | - | 0.069 | 0.087 | 0.128 | 0.068 |
| 5 | rs1065757 | 0.087 | 0.442 | 0.392 | 0.358 | 0.267 | 0.376 | - | 0.457 | 0.087 | 0.406 | - |
| 5 | rs3733801 | 0.057 | 0.112 | - | 0.074 | 0.228 | 0.137 | - | 0.202 | 0.061 | 0.124 | - |
| 5 | rs16872235 | 0.151 | 0.103 | - | - | 0.059 | - | 0.209 | 0.103 | 0.333 | 0.118 | 0.160 |
| 5 | rs2278492 | 0.192 | 0.333 | 0.397 | 0.375 | 0.426 | 0.329 | 0.171 | 0.474 | 0.157 | 0.279 | 0.204 |
| 5 | rs25640 | 0.212 | 0.478 | 0.474 | 0.431 | 0.421 | 0.455 | 0.119 | 0.457 | 0.055 | 0.446 | 0.078 |
| 5 | rs10069050 | 0.274 | 0.406 | 0.448 | 0.413 | 0.386 | 0.438 | 0.234 | 0.457 | 0.353 | 0.466 | 0.252 |
| 5 | rs702689 | 0.491 | 0.293 | 0.297 | 0.218 | 0.495 | 0.257 | 0.491 | 0.491 | 0.412 | 0.304 | 0.410 |
| 5 | rs10277 | 0.245 | 0.487 | 0.186 | 0.170 | 0.406 | 0.199 | 0.164 | 0.302 | 0.282 | 0.480 | 0.255 |
| 6 | rs3765310 | - | - | 0.056 | 0.101 | 0.106 | 0.131 | 0.069 | - | 0.051 | - | - |
| 6 | rs2766597 | 0.069 | - | - | - | - | - | 0.131 | - | 0.188 | - | 0.140 |
| 6 | rs12192544 | 0.057 | 0.201 | 0.102 | 0.142 | 0.327 | 0.120 | - | 0.147 | 0.064 | 0.230 | - |
| 6 | rs17601580 | - | 0.161 | - | - | 0.188 | - | - | 0.086 | 0.083 | 0.226 | - |
| 6 | rs7763565 | 0.085 | - | - | - | 0.074 | - | 0.196 | 0.216 | 0.141 | 0.059 | 0.153 |
| 6 | rs12530146 | 0.096 | - | 0.221 | 0.187 | 0.237 | 0.176 | 0.085 | - | 0.081 | 0.061 | 0.127 |
| 6 | rs9294445 | 0.387 | 0.446 | 0.092 | 0.096 | 0.406 | 0.075 | 0.400 | 0.319 | 0.381 | 0.451 | 0.381 |
| 6 | rs1051931 | - | - | - | - | - | - | - | - | 0.381 | 0.296 | 0.318 |
| 6 | rs2273566 | 0.353 | 0.095 | 0.438 | 0.424 | 0.124 | 0.486 | 0.315 | 0.161 | 0.240 | 0.116 | 0.366 |
| 6 | rs543580 | 0.066 | - | - | - | - | - | 0.064 | - | - | - | 0.061 |
| 6 | rs7744694 | 0.179 | - | - | - | - | - | 0.118 | - | 0.093 | - | 0.130 |
| 6 | rs11751765 | - | - | - | - | - | - | - | - | - | 0.059 | - |
| 6 | rs2076484 | 0.387 | - | 0.140 | 0.092 | 0.094 | 0.221 | 0.414 | 0.121 | 0.394 | 0.059 | 0.483 |
| 6 | rs2076485 | - | 0.234 | 0.180 | 0.202 | 0.307 | 0.164 | - | 0.233 | 0.093 | 0.188 | - |
| 6 | rs7744845 | 0.142 | 0.317 | 0.496 | 0.372 | 0.455 | 0.434 | 0.095 | 0.293 | 0.164 | 0.378 | - |
| 7 | rs1799805 | - | 0.054 | - | - | - | - | - | - | - | 0.104 | - |
| 7 | rs8286 | - | - | - | - | - | - | - | - | 0.198 | - | - |
| 7 | rs2303361 | 0.085 | 0.205 | 0.241 | 0.179 | 0.198 | 0.179 | - | 0.233 | - | 0.216 | 0.071 |
| 7 | rs12672205 | - | - | 0.146 | 0.139 | - | 0.077 | - | 0.114 | - | - | - |
| 7 | rs10266732 | 0.189 | - | - | - | - | - | 0.236 | - | 0.218 | - | 0.364 |
| 7 | rs9655651 | - | - | - | - | - | - | 0.060 | - | - | - | - |
| 7 | rs3918166 | 0.094 | - | - | - | - | - | 0.123 | - | 0.075 | - | 0.075 |
| 7 | rs4866 | - | - | - | - | - | 0.103 | - | - | - | - | - |
| 7 | rs17151689 | 0.075 | - | 0.110 | 0.069 | 0.079 | - | 0.128 | - | 0.224 | - | 0.109 |
| 7 | rs13306698 | - | - | 0.077 | 0.060 | - | 0.122 | - | - | - | - | - |
| 7 | rs1130499 | 0.471 | 0.333 | 0.471 | 0.491 | 0.260 | 0.478 | 0.486 | 0.456 | 0.330 | 0.304 | 0.452 |
| 7 | rs3752368 | - | - | 0.091 | 0.093 | - | 0.054 | - | - | - | - | - |
| 7 | rs1130496 | - | - | - | 0.419 | 0.370 | - | - | - | 0.187 | - | - |
| 8 | rs1058913 | 0.057 | 0.174 | 0.062 | - | 0.183 | - | - | 0.121 | - | 0.211 | - |
| 8 | rs2305129 | 0.231 | - | 0.131 | 0.151 | 0.119 | 0.084 | 0.268 | 0.060 | 0.260 | 0.078 | 0.235 |
| 8 | rs4871364 | 0.500 | 0.183 | 0.102 | 0.147 | 0.262 | 0.111 | 0.446 | 0.103 | 0.481 | 0.147 | 0.473 |
| 8 | rs17057255 | 0.077 | - | - | - | - | - | 0.151 | - | 0.158 | - | 0.092 |
| 8 | rs751141 | 0.066 | 0.095 | 0.265 | 0.206 | 0.114 | 0.235 | 0.132 | 0.164 | 0.194 | 0.059 | 0.085 |
| 8 | rs16880994 | 0.078 | - | - | - | - | - | 0.136 | - | - | - | 0.122 |
| 8 | rs17184326 | 0.094 | 0.156 | - | - | 0.084 | - | 0.205 | 0.086 | 0.221 | 0.132 | 0.197 |
| 8 | rs6558394 | 0.434 | 0.339 | 0.357 | 0.353 | 0.352 | 0.380 | 0.496 | 0.360 | 0.468 | 0.371 | 0.462 |
| 8 | rs16898023 | - | - | - | - | - | - | - | 0.054 | - | - | - |
| 8 | rs1346044 | 0.189 | 0.290 | 0.091 | 0.130 | 0.287 | 0.084 | 0.200 | 0.190 | 0.263 | 0.272 | 0.122 |
| 8 | rs1800391 | - | 0.071 | - | - | 0.109 | - | - | 0.078 | - | 0.084 | - |
| 8 | rs3802264 | - | - | 0.342 | 0.339 | 0.198 | 0.268 | - | - | - | - | - |
| 9 | rs6271 | - | 0.060 | - | - | - | - | - | 0.071 | - | - | - |
| 9 | rs3208406 | - | 0.071 | - | - | - | - | - | 0.078 | - | 0.152 | - |
| 9 | rs2480452 | - | 0.054 | 0.201 | 0.216 | 0.089 | 0.181 | - | 0.052 | - | 0.054 | - |
| 9 | rs573904 | 0.075 | 0.313 | 0.246 | 0.298 | 0.183 | 0.319 | 0.055 | 0.155 | 0.122 | 0.272 | 0.058 |
| 10 | rs1058930 | - | 0.067 | - | - | - | - | - | - | - | - | - |
| 10 | rs28371685 | - | - | - | - | - | - | - | - | 0.229 | - | - |
| 10 | rs2271904 | 0.179 | 0.068 | 0.326 | 0.212 | 0.257 | 0.264 | 0.250 | - | 0.211 | - | 0.171 |
| 10 | rs1886996 | 0.212 | 0.282 | - | - | - | 0.065 | 0.301 | 0.232 | 0.365 | 0.315 | 0.129 |
| 10 | rs814628 | - | 0.165 | 0.314 | 0.321 | 0.163 | 0.354 | - | 0.121 | 0.051 | 0.206 | - |
| 10 | rs17508082 | - | - | - | - | - | - | - | - | - | 0.083 | - |
| 11 | rs11042836 | - | 0.063 | - | - | - | - | - | 0.061 | - | 0.064 | - |
| 11 | rs2075291 | - | - | - | 0.051 | - | - | - | - | - | - | - |
| 11 | rs877711 | 0.170 | 0.156 | 0.099 | 0.083 | 0.188 | 0.124 | 0.168 | 0.259 | 0.224 | 0.064 | 0.187 |
| 11 | rs1801516 | - | 0.188 | - | - | 0.124 | - | - | 0.086 | - | 0.172 | - |
| 11 | rs2235000 | - | - | - | - | - | - | - | - | - | - | 0.112 |
| 11 | rs10891314 | 0.142 | 0.268 | 0.368 | 0.393 | 0.352 | 0.375 | 0.168 | 0.336 | 0.221 | 0.315 | 0.079 |
| 11 | rs11553595 | 0.115 | - | - | - | - | - | 0.093 | - | 0.064 | - | 0.120 |
| 11 | rs7948666 | 0.057 | - | - | - | - | - | - | - | - | - | 0.071 |
| 11 | rs28365927 | 0.106 | 0.152 | 0.142 | 0.106 | 0.350 | 0.165 | 0.055 | 0.105 | 0.176 | 0.157 | 0.078 |
| 11 | rs1050239 | 0.057 | 0.237 | 0.199 | 0.142 | 0.129 | 0.155 | 0.132 | 0.138 | 0.077 | 0.186 | 0.140 |
| 12 | rs2066828 | - | - | - | - | - | - | - | - | 0.196 | - | - |
| 12 | rs10507047 | 0.085 | 0.058 | 0.230 | 0.229 | 0.069 | 0.283 | 0.095 | 0.172 | 0.186 | 0.074 | 0.109 |
| 12 | rs5442 | - | 0.085 | - | - | - | - | - | - | - | - | - |
| 12 | rs1800973 | - | - | - | - | - | - | - | - | - | 0.066 | - |
| 12 | rs1047735 | 0.170 | 0.362 | 0.471 | 0.477 | 0.238 | 0.469 | 0.174 | 0.448 | 0.301 | 0.328 | 0.184 |
| 12 | rs9658403 | - | - | - | - | - | - | - | - | - | - | 0.054 |
| 12 | rs3751143 | 0.066 | 0.174 | 0.239 | 0.298 | 0.262 | 0.250 | 0.091 | 0.228 | 0.189 | 0.277 | 0.092 |
| 12 | rs7958311 | 0.283 | 0.196 | 0.452 | 0.427 | 0.089 | 0.341 | - | 0.285 | 0.174 | 0.206 | - |
| 12 | rs208294 | - | 0.455 | 0.371 | 0.463 | 0.411 | 0.443 | 0.204 | 0.319 | 0.403 | 0.490 | - |
| 12 | rs2287541 | 0.066 | 0.071 | - | - | 0.129 | - | - | 0.293 | - | 0.054 | - |
| 12 | rs12425042 | 0.066 | 0.143 | - | - | 0.064 | - | - | 0.095 | - | 0.172 | 0.054 |
| 12 | rs1177573 | 0.142 | - | - | - | - | - | 0.146 | - | 0.112 | - | 0.163 |
| 12 | rs5892 | - | - | - | - | 0.069 | - | 0.114 | - | 0.160 | - | 0.092 |
| 13 | rs9469 | 0.104 | 0.383 | 0.285 | 0.339 | 0.356 | 0.266 | 0.146 | 0.447 | 0.187 | 0.361 | 0.092 |
| 13 | rs17254379 | - | 0.149 | - | - | - | - | - | 0.112 | - | 0.172 | - |
| 13 | rs3742303 | - | - | 0.055 | - | 0.114 | 0.084 | - | - | - | - | - |
| 14 | rs11555803 | 0.085 | - | - | - | - | - | 0.082 | - | 0.104 | - | 0.058 |
| 14 | rs1885097 | 0.481 | 0.442 | 0.347 | 0.385 | 0.460 | 0.384 | 0.403 | 0.336 | 0.480 | 0.368 | 0.490 |
| 14 | rs3751501 | - | 0.063 | 0.088 | 0.174 | - | 0.117 | - | - | - | 0.054 | - |
| 14 | rs11620816 | 0.434 | 0.125 | 0.274 | 0.317 | 0.233 | 0.327 | 0.482 | 0.158 | 0.324 | 0.118 | 0.483 |
| 14 | rs17619 | 0.264 | - | - | - | 0.054 | 0.066 | 0.357 | - | 0.208 | 0.059 | 0.395 |
| 14 | rs11543947 | 0.066 | 0.099 | - | - | 0.066 | - | - | - | - | 0.069 | - |
| 14 | rs946616 | 0.151 | 0.098 | - | - | 0.069 | 0.078 | 0.188 | 0.053 | 0.140 | 0.108 | 0.259 |
| 14 | rs17104991 | - | - | - | - | - | - | - | - | 0.065 | - | - |
| 15 | rs11852361 | 0.151 | - | - | - | - | - | 0.092 | 0.055 | 0.082 | - | 0.058 |
| 15 | rs269868 | 0.198 | 0.054 | 0.056 | 0.060 | - | 0.058 | 0.191 | 0.164 | 0.391 | 0.059 | 0.133 |
| 15 | rs600753 | 0.302 | 0.433 | 0.252 | 0.275 | 0.470 | 0.389 | 0.364 | 0.207 | 0.442 | 0.490 | 0.219 |
| 15 | rs7168775 | 0.059 | - | - | - | - | - | 0.060 | - | - | - | - |
| 15 | rs3751593 | - | - | 0.067 | 0.060 | - | 0.111 | - | - | - | - | - |
| 15 | rs2061007 | 0.208 | 0.429 | 0.430 | 0.390 | 0.490 | 0.438 | - | 0.474 | - | 0.422 | 0.085 |
| 15 | rs2303580 | 0.387 | 0.188 | 0.369 | 0.399 | 0.218 | 0.385 | 0.318 | 0.319 | 0.164 | 0.255 | 0.412 |
| 15 | rs1912403 | - | 0.059 | - | - | - | - | - | - | - | 0.059 | - |
| 15 | rs4924595 | - | 0.058 | 0.197 | 0.262 | 0.069 | 0.214 | - | - | - | 0.078 | - |
| 15 | rs8179071 | - | - | - | - | 0.106 | - | - | - | - | - | - |
| 15 | rs16958445 | - | - | 0.135 | 0.156 | 0.104 | 0.173 | - | - | - | - | - |
| 15 | rs3743044 | - | - | 0.062 | - | 0.074 | 0.142 | - | - | - | - | - |
| 16 | rs13337489 | - | - | - | - | - | - | 0.100 | - | - | - | 0.058 |
| 16 | rs8176919 | 0.104 | - | - | - | - | - | - | - | 0.067 | - | 0.062 |
| 16 | rs8176927 | 0.144 | - | - | - | - | - | 0.092 | - | 0.106 | - | 0.119 |
| 16 | rs2791 | - | 0.058 | - | - | - | - | - | 0.052 | - | - | - |
| 16 | rs1800067 | - | - | - | - | - | - | - | - | - | 0.083 | - |
| 16 | rs17537869 | - | 0.112 | - | - | - | - | - | - | - | 0.074 | - |
| 16 | rs3810813 | - | - | 0.186 | 0.216 | - | 0.159 | - | 0.121 | - | 0.054 | - |
| 16 | rs12960 | 0.058 | 0.150 | - | - | 0.155 | - | - | 0.118 | - | - | - |
| 16 | rs10083789 | 0.123 | 0.317 | 0.058 | 0.050 | 0.094 | - | 0.114 | 0.259 | 0.218 | 0.279 | 0.099 |
| 16 | rs1978066 | - | - | 0.115 | 0.092 | - | 0.102 | - | - | - | - | - |
| 16 | rs9932912 | - | - | - | - | - | - | 0.083 | - | 0.098 | - | - |
| 17 | rs17741424 | - | 0.098 | - | - | 0.064 | - | - | 0.078 | - | 0.162 | - |
| 17 | rs13383 | - | - | - | - | - | - | - | - | 0.112 | - | - |
| 17 | rs3730043 | - | - | - | - | - | - | 0.056 | 0.053 | - | - | - |
| 17 | rs8065502 | 0.160 | 0.071 | - | - | - | - | 0.241 | 0.086 | 0.199 | 0.123 | 0.265 |
| 17 | rs1800315 | 0.170 | - | - | - | 0.064 | - | 0.164 | - | 0.081 | - | 0.174 |
| 17 | rs1800307 | - | - | 0.193 | 0.106 | - | 0.168 | - | - | - | - | - |
| 17 | rs3744215 | 0.198 | 0.286 | 0.381 | 0.417 | 0.277 | 0.420 | 0.376 | 0.149 | 0.308 | 0.352 | 0.298 |
| 17 | rs17651549 | 0.066 | 0.205 | - | - | 0.094 | - | - | 0.181 | - | 0.358 | - |
| 17 | rs11080149 | - | 0.143 | - | - | 0.059 | - | - | 0.095 | - | 0.078 | - |
| 17 | rs3730017 | 0.208 | - | - | - | - | - | 0.232 | 0.112 | 0.151 | - | 0.228 |
| 17 | rs155733 | 0.385 | 0.261 | - | - | 0.237 | - | - | - | - | 0.175 | - |
| 17 | rs3760460 | 0.144 | - | - | 0.091 | - | - | - | - | - | - | - |
| 17 | rs216195 | 0.311 | 0.330 | 0.365 | 0.422 | 0.406 | 0.332 | 0.341 | 0.164 | 0.232 | 0.211 | 0.299 |
| 17 | rs11650318 | 0.123 | 0.433 | 0.272 | 0.296 | 0.322 | 0.180 | 0.055 | 0.307 | 0.110 | 0.368 | - |
| 17 | rs17855672 | 0.123 | 0.442 | 0.280 | 0.306 | 0.325 | 0.188 | 0.068 | 0.298 | 0.113 | 0.373 | 0.056 |
| 17 | rs3744797 | - | - | 0.094 | - | - | 0.107 | - | - | - | - | - |
| 17 | rs9899177 | 0.255 | 0.360 | 0.173 | 0.181 | 0.238 | 0.084 | 0.182 | 0.431 | 0.292 | 0.348 | 0.194 |
| 18 | rs12968116 | 0.066 | 0.109 | - | - | 0.094 | - | - | 0.053 | 0.051 | 0.113 | - |
| 18 | rs3745078 | - | - | - | 0.078 | - | 0.063 | - | - | - | - | - |
| 19 | rs7412 | 0.125 | - | 0.090 | 0.070 | - | 0.053 | 0.052 | 0.054 | 0.061 | - | 0.083 |
| 19 | rs16994559 | 0.302 | - | - | - | 0.120 | - | 0.132 | - | 0.164 | 0.069 | 0.228 |
| 19 | rs8100856 | 0.151 | 0.165 | 0.409 | 0.427 | 0.134 | 0.443 | 0.159 | 0.353 | 0.103 | 0.108 | 0.116 |
| 19 | rs28399499 | 0.085 | - | - | - | - | - | 0.069 | - | - | - | 0.122 |
| 19 | rs8192709 | - | - | - | - | - | 0.066 | 0.081 | - | - | - | - |
| 19 | rs3765148 | 0.104 | 0.080 | 0.058 | - | - | 0.075 | 0.159 | 0.052 | 0.141 | 0.078 | 0.121 |
| 19 | rs602662 | 0.462 | 0.420 | - | - | 0.228 | - | 0.446 | 0.379 | 0.397 | 0.495 | 0.459 |
| 19 | rs1799817 | 0.226 | 0.237 | 0.415 | 0.376 | 0.352 | 0.376 | 0.246 | 0.224 | 0.266 | 0.216 | 0.225 |
| 19 | rs16994192 | - | - | - | 0.050 | - | - | - | - | 0.077 | - | - |
| 19 | rs11085147 | - | 0.107 | - | - | - | - | - | - | - | - | - |
| 19 | rs11564620 | 0.264 | 0.098 | - | - | 0.054 | - | 0.205 | 0.086 | 0.279 | 0.128 | 0.330 |
| 19 | rs2307282 | 0.160 | - | - | - | - | - | 0.114 | - | 0.135 | - | 0.136 |
| 19 | rs3761097 | - | - | 0.143 | 0.170 | - | 0.159 | - | - | 0.071 | 0.069 | - |
| 19 | rs1800472 | - | - | - | - | - | - | - | 0.054 | - | - | - |
| 19 | rs1027392 | 0.330 | 0.414 | - | 0.088 | 0.332 | 0.097 | 0.324 | 0.202 | 0.221 | 0.378 | 0.236 |
| 19 | rs3795003 | 0.066 | - | 0.416 | 0.385 | 0.069 | 0.412 | 0.065 | 0.218 | 0.119 | - | 0.062 |
| 20 | rs2273528 | 0.113 | - | 0.179 | 0.165 | - | 0.093 | 0.246 | 0.079 | 0.240 | - | 0.163 |
| 20 | rs6048066 | 0.385 | - | - | - | - | - | 0.417 | - | 0.253 | - | 0.486 |
| 22 | rs2076101 | 0.226 | 0.469 | 0.303 | 0.335 | 0.386 | 0.243 | 0.214 | 0.319 | 0.256 | 0.441 | 0.154 |
| 22 | rs8177832 | 0.302 | - | 0.085 | 0.069 | - | 0.071 | 0.339 | - | 0.176 | - | 0.459 |
| 22 | rs6151415 | - | 0.107 | - | - | - | - | - | - | - | 0.074 | - |
| 22 | rs362129 | 0.208 | 0.496 | - | - | 0.396 | 0.124 | 0.314 | 0.285 | 0.397 | 0.471 | 0.255 |
| 22 | rs2269383 | 0.104 | - | 0.168 | 0.162 | - | 0.089 | 0.123 | - | 0.151 | - | 0.095 |
| 22 | rs470117 | 0.179 | 0.469 | 0.453 | 0.450 | 0.307 | 0.491 | 0.100 | 0.457 | 0.106 | 0.456 | 0.082 |
| X | rs1050828 | 0.155 | - | - | - | - | - | 0.165 | - | - | - | 0.221 |
| X | rs2230036 | - | - | - | - | - | - | 0.103 | - | 0.067 | - | - |

^a^ “-” means MAF<0.05 in this Group.
