# Supplementary material for: Smaller Genetic Risk in Catabolic Process Explains Lower Energy Expenditure, More Athletic Capability and Higher Prevalence of Obesity in Africans
Source: PLoS One. 2011 Oct 10;6(10):e26027. doi: 10.1371/journal.pone.0026027 (PMC3189926; doi:10.1371/journal.pone.0026027)
Supplement: Table S4 — Analysis of Variance for Observations (ANOVA) of Num in human groups. (DOC) [file pone.0026027.s020.doc]

Table S4. Analysis of Variance for Observations (ANOVA) of Num in human groups ^a^

|  | Df | Sum Sq. | Mean Sq. | F value | Pr(>F) |
| --- | --- | --- | --- | --- | --- |
| Gender | 1 | 177 | 177.08 | 4.096 | 0.0432 * |
| Subpopulation | 2 | 3510 | 1755.19 | 40.60 | < 2.2$\times$10^-16^ *** |
| Group | 6 | 3034 | 505.71 | 11.70 | 9.07$\times$10^-13^ *** |
| Gender$\times$sub-  population | 2 | 105 | 52.39 | 1.211 | 0.2980 |
| Gender$\times$Group | 6 | 730 | 121.7 | 2.815 | 0.01005 * |
| Residuals | 1192 | 51532 | 43.23 |  |  |

*** The difference is greatly significant (P<<0.01).

* The difference is significant (P<0.05).

^a^ The data in African (ASW, LWK, MKK and YRI), East Asian (CHB, CHD and JPT) and European (CEU and TSI) are used for this analysis.
